# A population genomic resource for *Drosophila pseudoobscura*

**DOI:** 10.1093/g3journal/jkag210

**Published:** 2026-08-01

**Authors:** Yesbol Manat, Zhao Zheng, Camryn A Kritzell, Christopher A Gonzales, Richard P Meisel

**Affiliations:** Department of Biology and Biochemistry, University of Houston, Houston, TX 77204, United States; Department of Biology and Biochemistry, University of Houston, Houston, TX 77204, United States; Department of Biostatistics and Data Science, University of Texas Health Science Center, Houston, TX 77030, United States; Department of Biology and Biochemistry, University of Houston, Houston, TX 77204, United States; Department of Biology and Biochemistry, University of Houston, Houston, TX 77204, United States; Center for Depression Research and Clinical Care, University of Texas Southwestern, Dallas, TX 75390, United States; Department of Biology and Biochemistry, University of Houston, Houston, TX 77204, United States

**Keywords:** chromosomal inversions, population structure, Tajima's D, neo-sex chromosomes

## Abstract

*Drosophila pseudoobscura* is an historically important organism in evolutionary genetics, serving as a model system in studies of chromosomal inversions, speciation, sex chromosome evolution, and sex-ratio drive. However, previous population genetics analysis of *D. pseudoobscura* focused on individual chromosomes or used fragmented genome assemblies as a reference. To address these shortcomings, we generated a *D. pseudoobscura* population genomics resource consisting of newly sequenced genomes from 60 inbred lines sampled across the species' geographic range in North America. Using these data and a chromosome-scale reference genome, we examined patterns of nucleotide diversity and population structure across the chromosomes. We found no strong evidence of population structure on most chromosomes, consistent with prior results. In contrast, we identified population structure on the third chromosome, which we attributed to a well-characterized inversion polymorphism. We assigned individual third chromosome haplotypes to inversion arrangements, demonstrating how tests for population structure can be used to identify polymorphic chromosomal rearrangements. Tajima's *D* was negative across most of the genome, consistent with a recent population expansion. However, the distribution of genetic variation differed across third chromosome inversion arrangements in ways that were consistent with their hypothesized evolutionary histories, and we identified inter-arrangement genetic differentiation that could be attributed to the inversions suppressing genetic exchange. The population genomic data we have collected are publicly available and will support future research on evolutionary genetics.

## Teaser

The genomes of 60 isolates of *Drosophila pseudoobscura* were sequenced and analyzed. This species is a model organism for multiple areas in evolutionary genetics research, including chromosomal rearrangement, sex chromosomes, and speciation. This article presents the largest population genomic data set collected in this species. The analysis of the data demonstrates how population structure detection approaches can be used to identify polymorphic chromosomal inversions. The data presented will be valuable for future work on fundamental questions in population genetics.

## Introduction

Population genetics processes are greatly influenced by the structural organization of the genome. For example, chromosomal inversions suppress recombination in heterozygotes, allowing for the maintenance of linkage disequilibrium among coadapted complexes of alleles (i.e. supergenes) that would otherwise be broken apart by recombination (Wellenreuther and Bernatchez 2018; Berdan et al. 2023). Similarly, Robertsonian fusions between chromosomes can affect the recombination landscape, thereby shaping large-scale patterns of genetic diversity (Guerrero and Kirkpatrick 2014; Cicconardi et al. 2021; Yoshida et al. 2023). Because of these effects, both chromosomal inversions and fusions have been implicated as important contributors to the speciation process (White 1969; Rieseberg 2001; Faria and Navarro 2010). Fusions between sex chromosomes and autosomes may have especially pronounced effects because they cause ancestrally autosomal regions of the genome to become sex-linked, thereby dramatically shifting the effects of sex-specific selection pressures and hybridization between divergent karyotypes (Zhou and Bachtrog 2012; Bracewell et al. 2017; Wang et al. 2022).

*Drosophila pseudoobscura* is a classic model organism for exploring the effects of genomic rearrangements on genetic diversity, adaptation, and speciation. *D. pseudoobscura* harbors a rich inversion polymorphism on its third chromosome, which was one of the first population genetic data sets ever analyzed (Dobzhansky and Sturtevant 1938; Anderson et al. 1975, 1991). In addition, *D.pseudoobscura* has been widely used to study the role of chromosomal inversions in species divergence and reproductive isolation (Kulathinal et al. 2009; Fuller et al. 2019; Korunes et al. 2021). Furthermore, the Y and X chromosomes both have features that make them uniquely informative about sex chromosome evolution. Many genes that are Y-linked in other *Drosophila* species are now found on the diminutive chromosome 5 (“dot” chromosome, or element F) in *D. pseudoobscura*, and the species has a new Y chromosome not found beyond its closest relatives (Carvalho and Clark 2005; Larracuente et al. 2010; Bracewell and Bachtrog 2020). There was also a fusion between the ancestral *Drosophila* X chromosome and an autosome in the evolutionary lineage leading to *D. pseudoobscura*, which created a neo-X chromosome that is used as a natural experiment for studying the effects of X-linkage on molecular evolution (Sturgill et al. 2007; Vicoso et al. 2008; Meisel et al. 2010, 2012; Avila et al. 2014). The *D. pseudoobscura* X chromosome also harbors one of the first-ever described sex-ratio distortion systems, in which an X chromosome with multiple inversions causes the elimination of Y-bearing sperm (Sturtevant and Dobzhansky 1936; Novitski et al. 1965; Fuller et al. 2020). Moreover, *D. pseudoobscura* has a closelyrelated subspecies (*D. pseudoobscura bogotana*) and a sister species (*D. persimilis*), and this triad is a model system for speciation research, including investigating how chromosomal inversions, X-linked genes, and sex ratio distorters affect the speciation process (Fuller et al. 2019).

To improve the utility of *D. pseudoobscura* as an evolutionary genetics model organism, we sequenced and analyzed the genomes of 60 strains derived from independently isolated wild-caught female flies sampled across most of the species' range in North America (Fig. 1). Previously collected population genomic data sets from *D. pseudoobscura* either only sampled single chromosomes (Fuller et al. 2017, 2020) or performed genomewide analyses on a total of 31 unique strains (McGaugh et al. 2012; Samuk et al. 2020; Korunes et al. 2021). In addition, prior analyses did not use a chromosome-scale reference genome, which limited their ability to study the distribution of genetic diversity along each chromosome. Our genome-wide data have nearly twice the sample size of all previous genome-wide population genomics analyses, which we analyzed using a chromosome-scale reference. We used our data to identify single nucleotide polymorphisms (SNPs) across all 5 chromosomes. We then tested for signatures of genetic differentiation between third chromosome inversion arrangements and differences in evolutionary patterns between the X chromosome and autosomes. Our new dataset will allow for additional exploration of genome-wide genetic diversity in *D. pseudoobscura* to address other fundamental questions in evolutionary genetics.

**Fig. 1.**
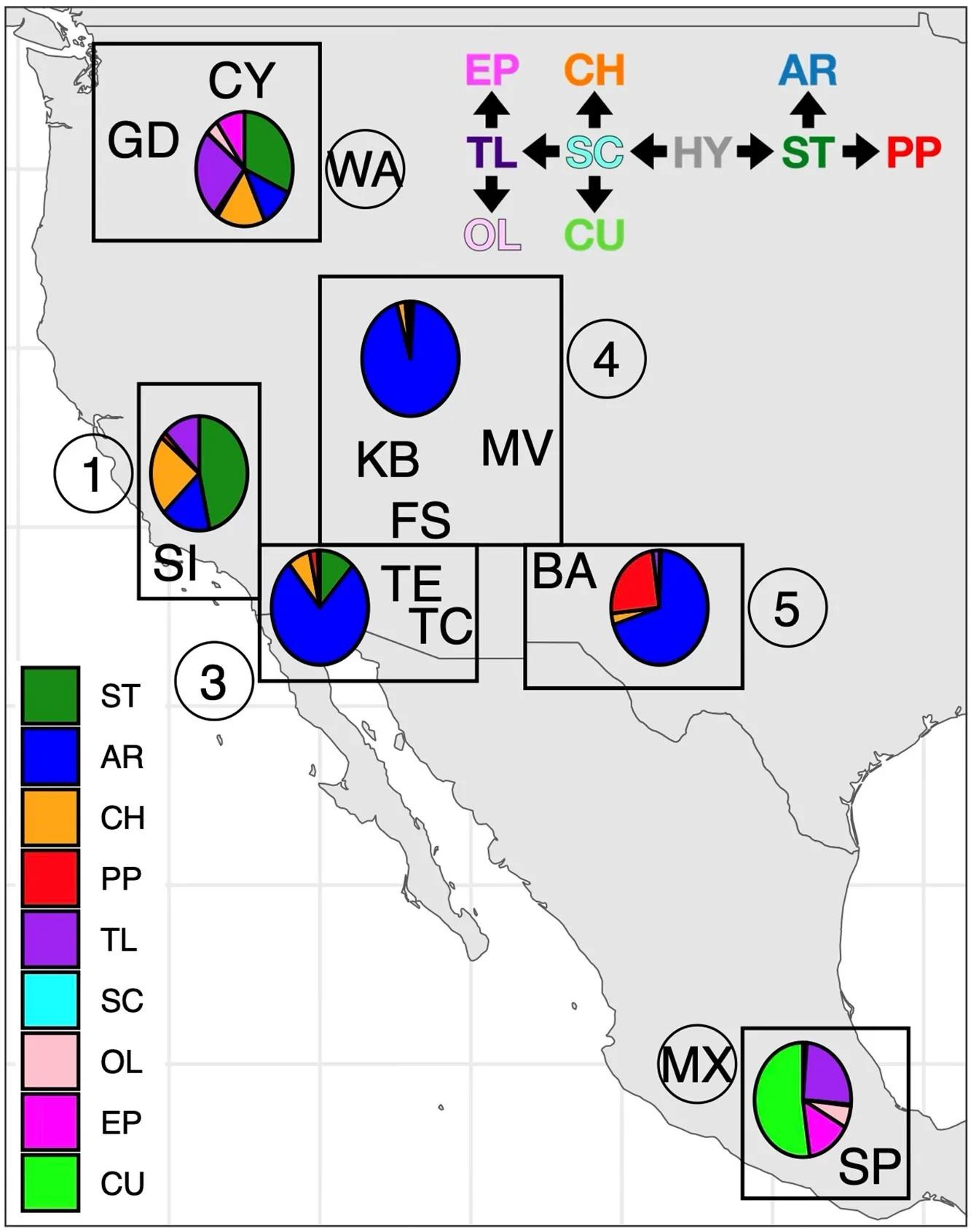
Frequencies of third chromosome arrangements in each sampled population. Each of the 10 sampled populations is shown with its two-letter code, with the position of the letters indicating the location of the population (Supplementary Table 1). Each population was assigned to 1 of 6 niches, which are represented by rectangles and labeled by numbers or letters within circles (WA, 1, 3, 4, 5, or MX). The frequencies of 9 third chromosome arrangements within each niche are represented by pie charts. The evolutionary relationships of the 9 arrangements (along with a hypothetical ancestral arrangement, HY) are shown in the top right of the figure. Each arrow represents a single chromosomal inversion event that differentiates adjacent arrangements in the network.

## Materials and methods

### Drosophila pseudoobscura genome organization

*Drosophila* genomes are organized into 5 large chromosome arms (Muller elements A–E) and a small “dot” chromosome with fewer than 100 genes (element F) (Muller 1940; Schaeffer et al. 2008). Element A is the ancestral X chromosome of the *Drosophila* genus. Element D was autosomal in the common ancestor of the genus, but it became fused with element A in the lineage leading to *D. pseudoobscura*. We refer to element D as the neo-X chromosome. Element C corresponds to chromosome 3 of *D. pseudoobscura*, which harbors a rich inversion polymorphism consisting of at least 30 different arrangements (Dobzhansky and Sturtevant 1938; Anderson et al. 1991; Wallace et al. 2011). Elements B and E correspond to *D. pseudoobscura* chromosomes 4 and 2, respectively (Schaeffer et al. 2008). *D. pseudoobscura* chromosome 5 consists of element F, which is highly heterochromatic and gene-poor across the *Drosophila* genus (Riddle and Elgin 2018), as well as ancestral Y chromosome genes that transposed to element F after the divergence of the *pseudoobscura* subgroup from sibling lineages (Larracuente et al. 2010; Bracewell and Bachtrog 2020). The *D. pseudoobscura* Y chromosome differs from the Y in other *Drosophila* species, likely because of processes related to the X-autosome fusion and Y-autosome transpositions (Carvalho and Clark 2005; Bernardo Carvalho et al. 2009). We did not include the Y chromosome in our analysis (because it is not present in the reference genome assembly), but we did include the ancestral X, neo-X, and all autosomes.

### Strain origins and sampling strategy

We sampled 64 strains of *D. pseudoobscura* that were each generated from single females collected from across the species range (Fig. 1). These isofemale strains were supplied by the *Drosophila* Species Stock Center. We have listed all of the information the stock center could provide about each strain in Supplementary Table 1, including the strain IDs. Each strain was sampled from one of 10 populations, with each population represented by 1 to 18 isofemale strains. Each of the 10 populations can be assigned to 1 of 6 ecological niches, which differ in climate and third chromosome arrangement frequencies (Dobzhansky 1948; Anderson et al. 1991; Schaeffer 2008). Only 60 samples were retained after filtering for sequencing coverage (see below). VCF files and detailed analysis methods are available in the Texas Data Repository (Manat and Meisel 2026).

### Sequencing methods and sample preparation

We extracted DNA from 5 to 10 individual flies from each of the 64 strains using a phenol:chloroform protocol (Sambrook and Russell 2006). The DNA was used to construct Illumina sequencing libraries using the Nextera XT DNA Sample Preparation Kit. All 64 libraries were sequenced on 4 separate runs of a NextSeq500 instrument at the University of Houston Seq-N-Edit core, with 150 bp paired-end reads.

### Variant calling and genotyping

We used a combination of BWA, GATK, and FreeBayes to determine the genotypes of each of the strains that we sequenced. We first mapped the sequencing reads to *D. pseudoobscura* genome assembly UCI_Dpse_MV25 (GCA_009870125; Liao et al. 2021) using BWA-MEM (Li 2013). Sequencing coverage statistics were calculated from BAM files using *samtools coverage* implemented in SAMtools v1.18 (Danecek et al. 2021), and mean read depth was estimated for each chromosome in each strain (Supplementary Fig. 1). We next used GATK v4.0.8.1 to identify duplicate reads, call variants, and recalibrate base calls based on high quality variants that we had identified (McKenna et al. 2010; Poplin et al. 2018; Van der Auwera and O’Connor 2020). We then used FreeBayes v1.3.7 (Garrison and Marth 2012) to genotype all 60 strains separately for each scaffold, providing population assignments with the—*- populations* option to partition the Bayesian inference model according to the populations we sampled (Fig. 1). To optimize runtime, the processes were carried out separately on 2 Mb segments of each chromosome (except for chromosome 5, which is only 1.88 Mb in length), and then we merged the VCFs using BCFtools concat (Danecek et al. 2021). Variants were filtered to retain only those with less than 50% missing data (−max-missing 0.5), with a minimum phred quality score of 30 (−minQ 30) and a read depth of 3 (−minDP 3) using VCFtools 1.18-GCC-12.3.0 (Danecek et al. 2011). This left us with 2,105,265 SNPs across the entire genome (Supplementary Table 2). We finally removed strains with more than 50% missing genotypes at variable sites, which excluded 4 strains and reduced the dataset from 64 to 60 strains (Supplementary Table 1).

### Population structure analysis

We used the data from the 60 retained strains to test for population structure using principal component analysis (PCA) and fastSTRUCTURE v1.0 (Raj et al. 2014). Prior to performing these analyses, we used VCFtools 1.18-GCC-12.3.0 (Danecek et al. 2011) to exclude variants with a minor allele frequency <0.05 (−maf 0.05) and a genotype call rate <95% (−max-missing 0.95), thereby removing rare and poorly genotyped variants. These filters substantially reduced the number of retained SNPs per chromosome, with an average retention rate of 39.5% across all chromosomes after filtering (Supplementary Table 2). We used the 831,808 remaining SNPs for population structure analysis. First, we performed PCA with *PLINK v2* (Chang et al. 2015), using linkage disequilibrium-pruned SNPs identified with the *–indep-pairwise 200 25 0.4* option.

We also used fastSTRUCTURE to identify how many population clusters (*K*) best explained the data for each chromosome. Our initial runs of fastSTRUCTURE used a simple (flat beta) prior, and we compared the fit of models 1 *≤ K ≤* 10. Estimating the most appropriate value of *K* from genotype data is challenging (Pritchard et al. 2000). We used the chooseK.py script in fastSTRUCTURE, which provides 2 separate approaches to identify the best-fitting *K* value to the data: one that maximizes marginal likelihood and another that best explains the structure in the data (Raj et al. 2014). If these 2 estimates of the best-fitting *K* values were equal for a chromosome, we used that value of *K* for that chromosome. However, if the 2 values of *K* differed for a given chromosome, we re-ran fastSTRUCTURE with a logistic prior using *K* values within the range of the 2 previously identified best-fitting values, following the recommended approach (Raj et al. 2014). We used the minimum *K* from the 2 approaches using the logistic prior as our *K* value for that chromosome. We then used the selected *K* for each chromosome to probabilistically assign each of the 60 strains to populations for each chromosome. We used these population assignments to assign some third chromosome haplotypes to inversion arrangements based on previously collected karyotype information (Fuller et al. 2016).

### Genetic variation across chromosomes

We calculated population genetic statistics on the data prior to filtering for minor allele frequencies and genotype call rates. These analyses were performed on data without the minor allele frequency filter applied because population genetics metrics are sensitive to the frequencies of rare alleles. To gain insights into the evolutionary forces acting on different parts of the genome, we separately analyzed data from intergenic, intronic, and synonymous sites, which may be subject to distinct selective pressures (Ellegren and Galtier 2016). To those ends, we assigned the 2,105,265 SNPs (Supplementary Table 2) to 1 of 4 functional categories (intergenic, intronic, synonymous, or missense) using SnpEff v5.2e (Cingolani, et al. 2012a, 2012b).

We also separately analyzed the third chromosome data within subsets of strains that we assigned to specific inversion arrangements. In these arrangement-specific analyses, we used previous mapping information to determine the locations of the chromosome 3 inversion breakpoints and to convert the coordinates of the reference genome (which carries an AR arrangement) into the order found in the TL arrangement (Wright 2023).

The following metrics were calculated within 100 kb windows with 10 kb step sizes. Tajima's *D* statistic (Watterson 1975; Tajima 1989) was calculated using VCFtools v0.1.16 and VCF-kit (Danecek et al. 2011; Cook and Andersen 2017) for intergenic, intronic, and synonymous sites across the *D. pseudoobscura* genome. Tajima's *D* measures the difference between the average pairwise diversity (*π*) and the number of segregating sites (*Θ*_W_). We therefore also separately calculated *π* and *Θ*_W_ using VCFtools v0.1.16 across the same sites as Tajima's *D*. Lastly, Weir and Cockerham's F_ST_ (1984) was calculated for intergenic sites between third chromosome arrangement haplotypes using the weighted method implemented in VCFtools v0.1.16. All calculations were performed within the same window and step sizes to ensure comparability across statistics.

## Results

We analyzed genome sequence data collected from 60 *D. pseudoobscura* strains that were sampled from 10 populations across the species range in North America (Fig. 1) (Supplementary Table 1). Mean sequencing depth per strain was 61.5, ranging from 5.8 to 425 across chromosomes and strains (Supplementary Fig. 1). We used these sequencing data to call variants and genotype each of the strains.

### Chromosome-specific population structure

We tested for population stratification in our data using fastSTRUCTURE (Raj et al. 2014). To this end, we identified how many population groups (*K*) could best explain the genetic diversity on each chromosome. We first used a simple (flat beta) prior and *K* values ranging from 1 to 10, assessing model fit using 2 criteria (see Methods). The 2 criteria for the best-fitting *K* value were consistent for chromosome 2 (*K* = 2) and chromosome 3 (*K* = 5) (Supplementary Table 3). However, for chromosome 2, nearly all (56/60) of the strains had >99.999% assignment to one of the populations, and an additional strain had >95% assignment to that same population (Fig. 2a). Only 3 strains had >5% assignment to a second population, and only 2 of those had >35% assignment to the second population. Three of the 4 strains with the highest proportional assignment to the second population were derived from females sampled from Mexico (SP population), but most of the Mexico-derived strains had >99% assignment to the first population. The fourth strain in the second population was from Niche 5 (BA population). The SP and BA populations were on the southern end of our sampling area (Fig. 1). Consistent with the fastSTRUCTURE results, PCA of chromosome 2 showed little evidence of population structure (Supplementary Fig. 2). The first principal component (PC1) explained 13.47% variation, with one sample each from the BA and SP populations separated from the main cluster along the PC1 axis. Also consistent with fastSTRUCTURE, the majority of samples from the BA and SP populations clustered with the other strains. We thus conclude that, while there was some evidence for population structure along the north-south axis of the species range for chromosome 2, the evidence was weak.

**Fig. 2.**
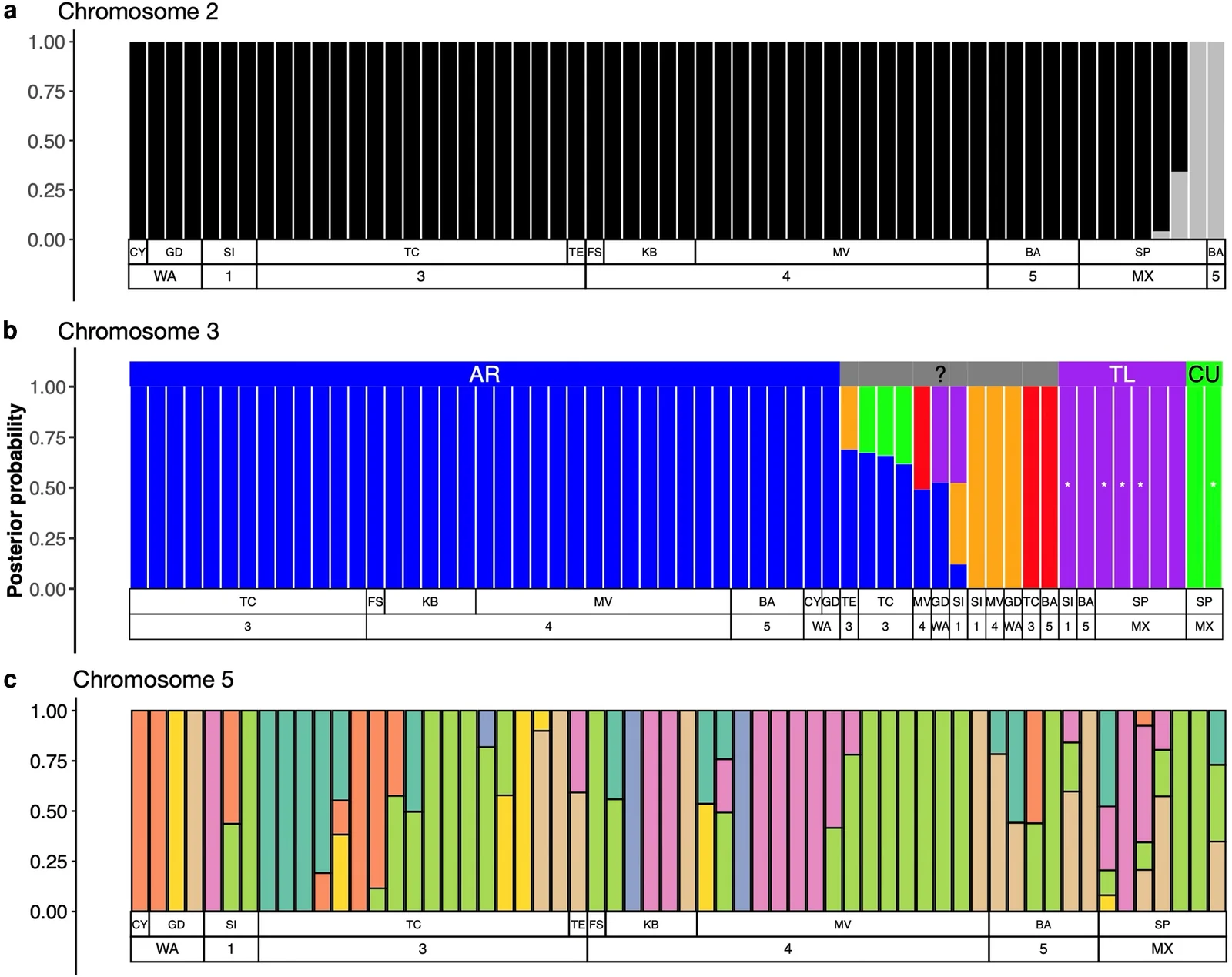
Population structure based on posterior probabilities of assignment to genotype clusters. Each bar represents one of the 60 strains, and the height of each color segment within a bar reflects the probability of assignment to a particular genetic cluster. The 2 rows below each graph indicate the population from which each strain was sampled and the associated ecological niche (see Fig. 1). a) Bars show the probabilistic assignment of individuals based on chromosome 2 genetic variation to *K* = 2 populations (black and gray). b) Probabilistic assignment of individuals to *K* = 5 populations is based on chromosome 3 genetic variation, where populations likely correspond to third chromosome inversion arrangements. Blue bars correspond to the AR-assigned strains, purple bars correspond to the TL-assigned strains, and green bars correspond to the CU-assigned strains. The red and orange colors indicate additional clusters of uncertain or mixed ancestry. Asterisks show individuals that were karyotyped for specific chromosomal arrangements (Fuller et al. 2016). c) Bars show the probabilistic assignment of individuals based on chromosome 5 genetic variation to *K* = 5 populations, each colored differently.

We hypothesized that the population structure on chromosome 3 could be explained by the third chromosome inversion polymorphism (Fig. 2b). The frequencies of the third chromosome inversion arrangement differ across the 6 ecological niches from which we have sampled our strains (Schaeffer 2008) (Fig. 1). To evaluate if the inversion polymorphism explains the chromosome 3 population structure, we attempted to assign each of the 5 populations to third chromosome inversion arrangements. The most common population (blue in Fig. 2b) likely corresponds to the AR arrangement because AR is at high frequencies in niches 3, 4, and 5, where those strains were sampled (Fig. 1). Individuals with >99% posterior probability of ancestry in the blue population were therefore assigned an AR genotype. The second most common population (purple in Fig. 2b) likely corresponds to the TL arrangement because 4 of those strains have been karyotyped as TL homozygotes (Fuller et al. 2016). Additional individuals were assigned the TL genotype if they had >99% posterior probability assignment to the purple population. Two samples likely corresponded to the CU arrangement, one of which was previously karyotyped as homozygous for the CU arrangement (Fuller et al. 2016). These strains had >99% posterior probability of assignment to the green population (Fig. 2b). The remaining 12 samples could not be unambiguously assigned to one of the arrangement genotypes because we lacked a karyotyped individual or they had mixed population assignments. Some strains with mixed assignment may be segregating for multiple chromosomal arrangements (despite being inbred). Alternatively, genetic exchange between inversion arrangements may have created haplotypes with mixed arrangement assignments (Schaeffer et al. 2024). Additional work is required to test these hypotheses for strains without an arrangement assignment.

PCA revealed a similar pattern of population structure on chromosome 3 as we found with fastSTRUCTURE (Supplementary Fig. 3). PC1 explained 18.97% of the genetic variation, and strains assigned to the AR arrangement in our fastSTRUCTURE analysis were clustered separately from the remaining strains along the PC1 axis. Strains assigned to the TL and CU arrangements were also clustered together along most PC axes, but the 2 arrangements were separated from each other by PC8. Principal components 2 to 7 individually explained 7.8% to 10.7% of the total variance, but they did not show clear clustering patterns by any of our arrangement assignments (Supplementary Fig. 3).

For chromosomes X, 4, and 5, the best fitting *K* values differed between the 2 criteria we used to evaluate model fit when we used a simple prior. We therefore re-ran fastSTRUCTURE with a logistic prior for these chromosomes. The optimal *K* for chromosome 5 remained >4 when we used a logistic prior, demonstrating strong evidence for population structure that may be complex because we failed to converge on a single best fitting *K* (Supplementary Table 3). We could not detect any association between the geographic origins of the strains and population assignments for chromosome 5 (Fig. 2c). However, PC1 for chromosome 5 explained 20.1% of variation, and it appeared to separate the samples from Niche 3 (collected in Arizona) and most samples from other populations (Supplementary Fig. 2). Below, we further explore the genetic variation on chromosome 5.

Chromosomes X and 4 had the least evidence for population structure. Using a logistic prior, at least one of our model selection criteria identified *K* = 1 as the best fitting value for the X and fourth chromosomes (Supplementary Table 3). The lack of a consistent signal of *K* > 1 suggests no population structure on chromosomes X and 4. However, PCA of chromosomes 4 and X identified outlier samples that resembled those we detected for chromosome 2. In particular, PC1 explained 13.08 and 13.34% of the variation on chromosomes 4 and X, respectively, and one strain each from the BA and SP populations clustered separately from other strains (Supplementary Fig. 2). These patterns provide additional evidence for a small amount of population structure along the north-south axis of the species range.

### Chromosome-specific patterns of genetic diversity

We next tested for departures from neutral expectations of heterozygosity on each chromosome using Tajima's *D* (Tajima 1989). The lack of strong evidence for population structure across most of the genome allowed us to treat our samples as if they came from a single population in these analyses. We calculated Tajima's *D* within 100 kb sliding windows across each chromosome for 3 classes of sites—intergenic, intronic, or synonymous. For the third chromosome, we also calculated *D* within individual inversion arrangements.

Consistent with previous observations (e.g. Fuller et al. 2017), we observed that *D* < 0 across *D. pseudoobscura* chromosomes (Fig. 3a; Supplementary Figs. 4 and 5). Regardless of which sites we analyzed, the average *D* for chromosomes X, 2, 3, and 5 fell between −0.9 and −1.0. However, chromosome 5 had a substantially higher *D* than the other chromosomes (Fig. 3a). We also observed distinct troughs (*D* ≪ −2) and peaks (*D* ≫ 0) in Tajima's *D* across all chromosomes, indicating local reductions or elevations in heterozygosity. The positions of these features varied among intergenic, intronic, and synonymous sites (Fig. 3a; Supplementary Figs. 4 and 5), with no troughs overlapping all 3 categories.

**Fig. 3.**
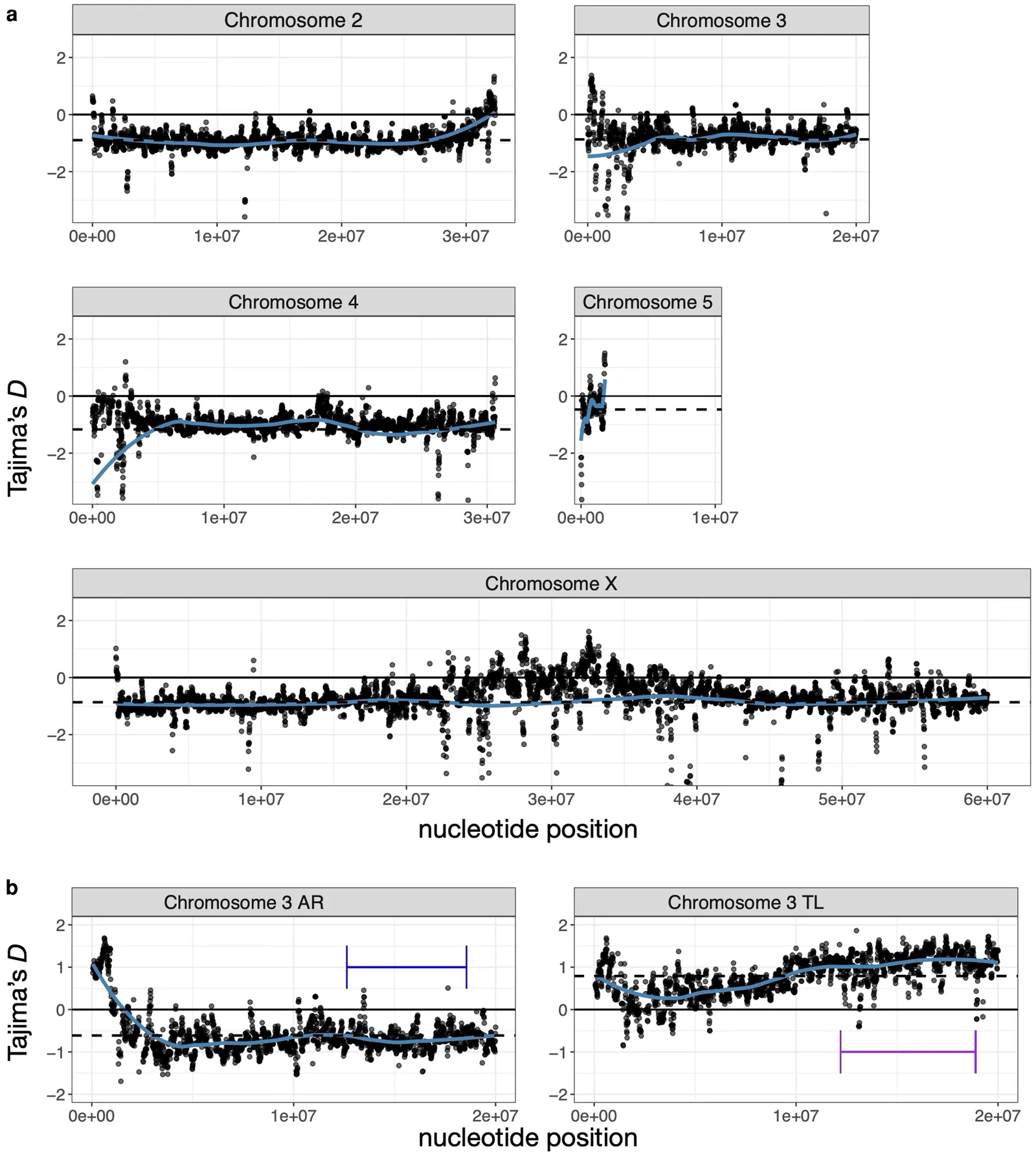
Tajima's *D* across windows on each *Drosophila pseudoobscura* chromosome. Dots show Tajima's *D* values for 100 kb sliding windows (with a 10 kb step) using intergenic sites across each of the 5 chromosomes arms. The solid lines show the generalized additive models with integrated smoothness estimation (GAMs) for each chromosome. The dashed lines show the mean Tajima's *D* values for each chromosome. a) Tajima's *D* was calculated using data from each chromosome based on all 60 strains in the dataset. b) Tajima's *D* was calculated using third chromosome genetic variation from AR-assigned or TL-assigned strains. Horizontal lines with t-bars indicate the positions of the AR and TL inversions.

To further investigate regions potentially shaped by positive selection, we focused on troughs, defined as genomic windows with Tajima's *D* < −2. This threshold is commonly used to detect regions with significantly reduced diversity, often interpreted as evidence of recent selective sweeps (Tajima 1989; Nielsen et al. 2005). Given the 100 kb window size that we used, these troughs often spanned broad genomic regions, meaning that genes within or overlapping these windows were likely affected by the same evolutionary pressures influencing local genetic variation. We specifically searched for genes located within overlapping troughs identified in at least 2 functional site classes. We found such overlapping troughs between intergenic and intronic sites in 6 windows on chromosome 3 and 6 windows on chromosome 4 (Supplementary Table 4). However, we did not identify any functional annotations that were significantly enriched or depleted among the genes within these regions. In addition, none of the genes located in these overlapping troughs matched previously identified candidate targets of selection in the *D. pseudoobscura* genome (Fuller et al. 2017).

We next examined the 2 measures of genetic diversity that are used to calculate Tajima's *D*, average pairwise diversity between haplotypes (*π*) and number of segregating sites (*Θ*_W_). Estimates of *π* and *Θ*_W_ varied across the lengths of chromosomes, largely in similar patterns regardless of which class of sites we used (Fig. 4; Supplementary Figs. 6–10). We observed reductions in both *π* and *Θ*_W_ at one or both ends of chromosomes 2 and 4, as well as near the center of the X chromosome, likely reflecting the effects of suppressed recombination near centromeres or telomeres (Langley et al. 2012; Mackay et al. 2012). The most striking pattern emerged on chromosome 3, where genetic variation was dramatically lower on the left end of the chromosome and rose substantially higher in the rightmost two-thirds. The pattern on chromosome 3 is likely attributable to sampling different inversion arrangements (see below). However, because *π* and *Θ*_W_ were similarly affected across the entire genome, Tajima's *D* did not vary as dramatically as its component genetic diversity metrics (Fig. 3a).

**Fig. 4.**
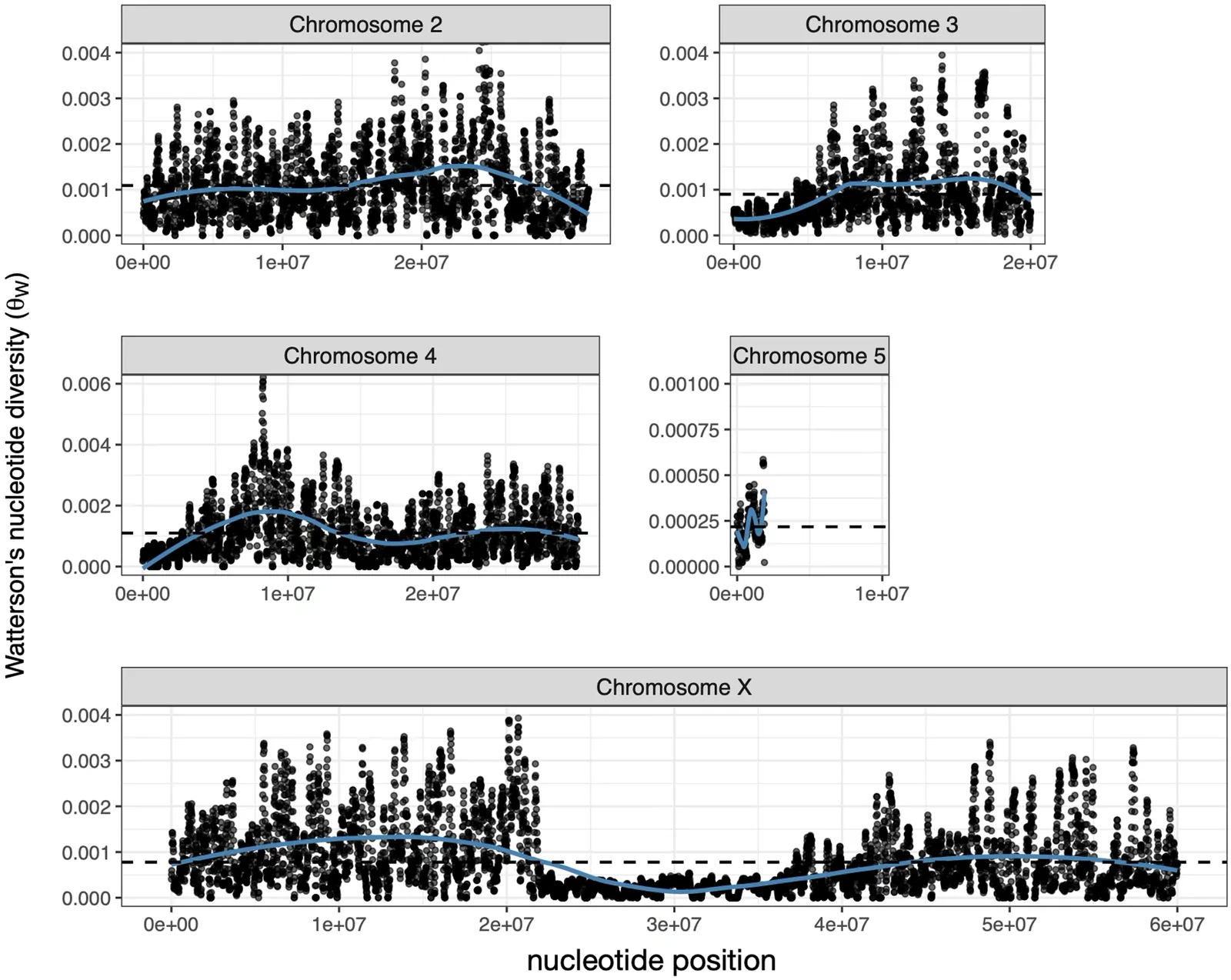
Segregating genetic variation (Watterson's *Θ*_W_) across windows on each *Drosophila pseudoobscura* chromosome. Dots show *Θ*_W_ for 100 kb sliding windows (with a 10 kb step) using intergenic sites across each of the 5 chromosomes arms. The solid lines show the generalized additive models with integrated smoothness estimation (GAMs) for each chromosome. The dashed lines show the mean *Θ*_W_ for each chromosome.

### Genetic variation is affected by chromosome 3 inversions

We further investigated how the inversions affect genetic variation on chromosome 3. Most of the chromosome 3 inversions are located on the right side of the chromosome (Fuller et al. 2017), including the inversions that gave rise to the AR and TL arrangements that we hypothesize make up the majority of our samples (Fig. 2). As described above, we detected elevated genetic variation on the right side of chromosome 3 (Fig. 4; Supplementary Figs. 6-10). Elevated *π* is consistent with differentiation between arrangement haplotypes within the inverted regions (Navarro et al. 2000; Machado et al. 2007; Noor et al. 2007), and elevated *Θ*_W_ is consistent with sampling arrangement-specific alleles from multiple arrangements (Fuller et al. 2017). We therefore hypothesized that the inversions affected genetic variation on chromosome 3 within strains and differences between arrangement haplotypes.

To determine how the chromosome 3 inversions affected genetic variation, we separately analyzed data from strains assigned to the AR arrangement and strains assigned to the TL arrangement, based on the fastSTRUCTURE analysis (Fig. 2b). Consistent with the pattern in our complete dataset, *D* < 0 along the entire length of chromosome 3 for AR-assigned strains (Fig. 3b; Supplementary Fig. 11). In contrast, *D* > 0 for TL-assigned strains across the entire third chromosome. We converted the chromosome 3 coordinates into the order found in the TL arrangement, and we observed the highest *D* values for TL individuals on the right half of the third chromosome, regardless of whether we used intergenic, intronic, or synonymous sites to calculate *D* (Fig. 3b; Supplementary Fig. 11). The elevated *D* coincides with the location of the inversion that gave rise to the TL arrangement (Fig. 3b).

We then used F_ST_ for intergenic sequences as a measure of differentiation between chromosomal arrangements. Comparing AR-assigned strains against TL-assigned strains, we observed dramatically higher F_ST_ within and adjacent to the location of the inversion that gave rise to the AR arrangement (Fig. 5a). We detected a less pronounced elevation in the same region of chromosome 3 when we compared AR-assigned individuals against individuals not assigned to either AR or TL (Fig. 5b). Similar patterns were observed when we compared TL against AR or all other individuals using the TL coordinates for chromosome 3 (Fig. 5c-d). These results provide a consistent signal that the chromosomal inversions affect genetic differentiation between third chromosome arrangements.

**Fig. 5.**
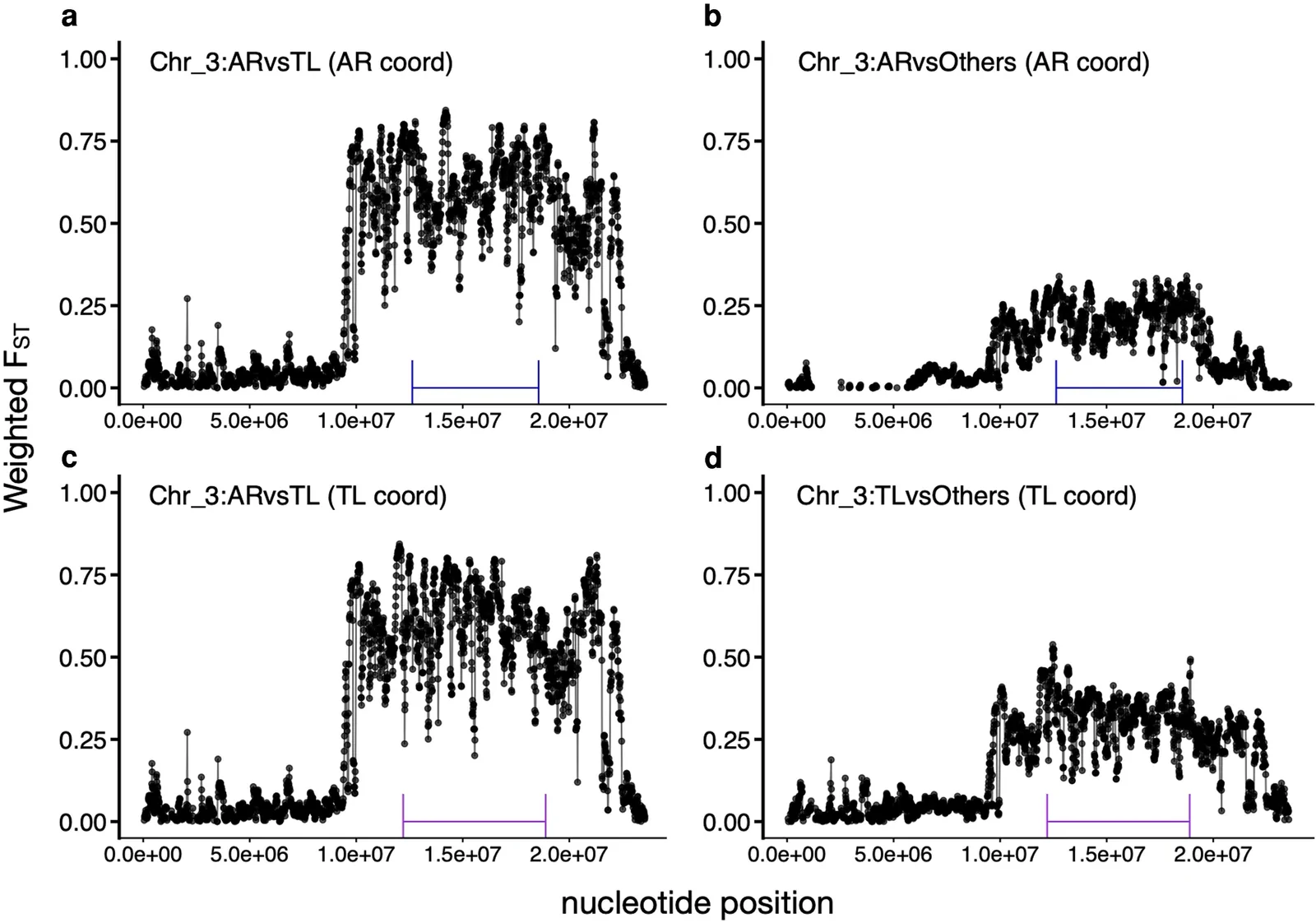
Arrangement-specific patterns of genetic differentiation across chromosome 3. Dots show F_ST_ for 100 kb sliding windows (with a 10 kb step) using intergenic sites across chromosome 3. Horizontal lines with t-bars indicate the positions of the AR and TL inversions. a) F_ST_ was calculated between AR-assigned and TL-assigned strains, and the AR coordinates were used for the nucleotide positions. b) F_ST_ was calculated between AR-assigned strains and strains not assigned to either AR or TL, with the AR coordinates for nucleotide positions. c) F_ST_ was calculated between AR-assigned and TL-assigned strains, and the TL coordinates were used for the nucleotide positions. d) F_ST_ was calculated between TL-assigned strains and strains not assigned to either AR or TL, with the AR coordinates for nucleotide positions.

## Discussion

We generated a population genomics dataset consisting of 60 *D. pseudoobscura* strains that were independently derived from flies sampled from natural populations across the species range (Fig. 1). We found very little evidence for population structure across most of the genome (Fig. 2a; Supplementary Table 3), but Tajima's *D* < 0 suggests that the species likely experienced a recent expansion in size (Fig. 3a). Our inferences about population structure and growth from our genome-wide data are consistent with previous findings based on small numbers of loci (Schaeffer and Miller 1992; Hamblin and Aquadro 1999; Kovacevic and Schaeffer 2000; Machado et al. 2002; Schaeffer et al. 2001; 2003). In contrast to most of the genome, we identified strong evidence for population structure on chromosome 3 (Fig. 2b), which is likely attributable to the well-documented inversion polymorphism (Anderson et al. 1991; Fuller et al. 2019). We furthermore found that patterns of genetic variation differed between third chromosome inversion arrangements (Figs. 3b and 4), while divergence between haplotypes appeared to be affected by the inversions (Fig. 5).

We observed very little evidence for population structure across most of the genome. For example, on chromosomes 4 and X, we could not reject a model with *K* = 1 (Supplementary Table 3). These findings are consistent with previous analyses of individual loci that found no evidence for population structure in *D. pseudoobscura* (Schaeffer and Miller 1992; Kovacevic and Schaeffer 2000). In contrast, we found evidence that *K* = 2 for chromosome 2 (Fig. 2a), suggesting some population structure that may be localized to portions of the genome. We detected a similar moderate population structure using PCA for chromosomes 2, 4, and X (Supplementary Fig. 2). The secondary population we identified in these analyses is associated with a small number of strains sampled from the southernmost collection sites in our data set. These analyses are suggestive that there may be slight restrictions to north-south gene flow across the species range.

We detected substantially more evidence for population structure on chromosome 5 than most of the other chromosomes (Fig. 2c). *D. pseudoobscura* chromosome 5 differs from the rest of the genome in 2 important ways: first, it consists of element F (i.e. the “dot” chromosome), and, second, it contains many genes that are found on the ancestral *Drosophila* Y chromosome (Larracuente et al. 2010). *Drosophila* element F is a small chromosome with fewer than 100 genes, and it has a heterochromatin environment that differs from most of the rest of the genome (Riddle and Elgin 2018). Element F is also the ancestral X chromosome of flies, and it evolved into an autosome in the lineage leading to the *Drosophila* genus (Vicoso and Bachtrog 2013). Previous work found that Tajima's *D <* 0 across much of *D. pseudoobscura* chromosome 5 (Larracuente and Clark 2014), consistent with our results (Fig. 3a). Chromosome 5 was also previously shown to have lower pairwise nucleotide diversity than much of the rest of the genome (Chang and Larracuente 2017), but we observed that both *π* and *Θ*_W_ were lower on chromosome 5 than on all other chromosomes (Fig. 4; Supplementary Figs. 6–10). Because *Θ*_W_ is lower on chromosome 5, it has a less negative Tajima's D when compared to other chromosomes (Fig. 2). The unique distribution of genetic variation on chromosome 5, along with an extremely low recombination rate (Wang et al. 2002; Arguello et al. 2010), may be responsible for the evidence of population structure that we discovered (Fig. 2c). Further work is required to investigate the causes of the unique genetic variation on chromosome 5.

The evidence for population structure on the third chromosome (Fig. 2b) can likely be explained by the inversion polymorphism that segregates in natural populations of *D. pseudoobscura* (Dobzhansky and Sturtevant 1938; Anderson et al. 1975, 1991). Most of the strains we sampled probably carry the AR arrangement because this is the most common third chromosome type found in the populations from which these strains were derived (Dobzhansky 1944). We could assign some of the remaining strains to 1 of 2 additional third chromosome arrangements (TL and CU) based on previous karyotyping of flies from these same isofemale lines (Fuller et al. 2016). We could not unambiguously assign the remaining strains to arrangements, but we hypothesize that some carry the PP (likely red) and CH (likely orange) arrangements based on their abundance in the populations from which they were sampled (Figs. 1 and 2b). Our results provide, to the best of our knowledge, the first example of using STRUCTURE or fastSTRUCTURE to identify inversion polymorphisms in natural populations.

The lack of pronounced population structure allowed us to treat most of the genome as if it came from a single population for the purpose of testing for deviations from equilibrium. Population structure affects neutral variation in ways that can resemble some forms of selection and dampen other signals of selection (Charlesworth et al. 1997; Nordborg and Innan 2003; Wright et al. 2003; Moeller et al. 2007). Because there was such weak evidence for population structure, we examined alternative explanations for the non-neutral patterns of genetic variation that we observed.

Without strong evidence for structure on chromosomes X, 2, and 4, we are left with 3 interpretations of genome-wide Tajima's *D* < 0 in *D. pseudoobscura* (Fig. 3a). The first 2 interpretations involve the effects of natural selection, specifically background selection or selective sweeps. If background selection against deleterious mutations and/or the adaptive fixation of beneficial alleles are sufficiently common, the majority of the genome may be genetically linked to sites under selection causing a genome-wide departure from neutral expectations (Sella et al. 2009). There is indeed evidence that background selection is pervasive in the *Drosophila melanogaster* genome (Comeron 2014). However, the effects of background selection are expected to differ depending on the extent of selective constraint on linked sites (Schrider 2020). We observed similar patterns of *D* < 0 in at intergenic, intronic, and synonymous sites (Fig. 3a; Supplementary Figs. 4 and 5), suggesting that background selection is not responsible for the departure from neutrality. In addition, prior studies of patterns of diversity in the *D. pseudoobscura* genome demonstrated that levels of genetic variation are not correlated with recombination rates in ways that would be consistent with pervasive selection driving departures from neutrality (Kulathinal et al. 2008). We therefore conclude that *D* < 0 genome-wide in *D. pseudoobscura* is unlikely to be caused by selective sweeps or background selection.

The third interpretation of *D* < 0 is that there has been a recent increase in the effective population size of *D. pseudoobscura*. We observed that Tajima's *D* < 0 across the genome (Fig. 3a), which is consistent with previous studies of individual *D. pseudoobscura* loci or chromosomes (Hamblin and Aquadro 1999; Kovacevic and Schaeffer 2000; Schaeffer et al. 2001; Machado et al. 2002; Schaeffer 2002; Schaeffer et al. 2003; Larracuente et al. 2010; Fuller et al. 2017). When *D* < 0 across the entire genome, it suggests that demographic history (e.g. population expansion) is responsible for the pattern (Hartl and Clark 2007).

Additional evidence that *D* < 0 is caused by an increase in population size comes from X-autosome contrasts. Both arms of the *D. pseudoobscura* X chromosome (i.e. the ancestral X and neo-X) have average Tajima's *D* values that resemble the major autosomes (Fig. 3a). If selective sweeps of new recessive mutations were responsible for *D* < 0, we would expect those effects to be greater on the X than the autosomes (Harris et al. 2024). The greater effect on X-linked genes is expected because recessive beneficial mutations are exposed to selection on the X in males, who only have a single X chromosome, while autosome recessive mutations are masked (Charlesworth et al. 1987; Meisel and Connallon 2013). Not only do we not observe X-autosome differences in Tajima's *D* but we also do not observe differences between the neo-X and ancestral X arms (Fig. 3a). The lack of X-autosome differences in Tajima's *D* is consistent with demographic effects (population expansion) driving the genome-wide *D* < 0.

Even more evidence that demographic history, as opposed to selection, drives *D* < 0 in *D. pseudoobscura* comes from X-autosome comparisons of *Θ*_W_ and *π*. In contrast to Tajima's *D*, both *Θ*_W_ and *π* for the X chromosome are lower than the autosomes (Fig. 4; Supplementary Figs. 6–10). *Θ*_W_ and *π* each scale with the product of the effective population size (*N_e_*) and mutation rate (Watterson 1975; Tajima 1989). X-autosome differences in *Θ*_W_ and *π* could thus be caused by differences in the population-level mutation rates and/or differing *N_e_*. A higher autosomal mutation rate can arise because of female-biased transmission of the X chromosome and higher mutation rates in the male germline, but evidence for this phenomenon in *Drosophila* is mixed (Bauer and Aquadro 1997; Bachtrog 2008; Wang et al. 2023). Alternatively, *N*_e_ of the X chromosome (*N*_eX_) is expected to be 75% of *N*_e_ of the autosomes (*N*_eA_) because there are 3/4 as many X chromosomes transmitted between generations, assuming equal numbers of breeding males and females (Singh et al. 2007; Vicoso and Charlesworth 2009). Both the mutational and population size effects would give rise to lower *Θ*_W_ and *π* on the X chromosome as compared to the autosomes, but the magnitude of the effects should be larger if under the population size effect (Ellegren 2007). We observed X/autosome ratios in *Θ*_W_ and *π* on the scale of 75% (Fig. 4; Supplementary Figs. 6–10), consistent with the X-autosome differences being caused by *N*_eX_ < *N*_eA_. Once again, this is a demographic explanation for patterns of genetic diversity, suggesting that demographic history best explains the genome-wide average deviation from equilibrium. This supports our hypothesis that population expansion is the best explanation for Tajima's *D* < 0 genome-wide.

Our conclusion about the prevailing effect of population expansion on genetic variation in *D. pseudoobscura* does not exclude selection also affecting genetic variation. For example, we observed distinct troughs (*D* ≪ −2) and peaks (*D* ≫ 0) in Tajima's *D* across all chromosomes, but the positions of these features varied among intergenic, intronic, and synonymous sites (Fig. 3a; Supplementary Figs. 2 and 3). No troughs overlapped all 3 categories, suggesting that selection may affect local patterns of genetic variation in a site-class-specific manner. Troughs in *D* could indicate loci that experienced a recent selective sweep, which could be explored in future work.

Additional evidence of selection comes from comparisons of polymorphism between third chromosome haplotypes assigned to different inversion arrangements. The majority of strains in our dataset were assigned to the AR arrangement (Fig. 2), and Tajima's *D* < 0 on the third chromosome for those AR-assigned strains (Fig. 3). There was also a moderate elevation of *D* near the breakpoints of the AR inversion in those AR-assigned strains (Fig. 3b), consistent with previous results (Fuller et al. 2017). AR is one of the youngest (most recently derived) third chromosome arrangements, and it has recently increased in frequency across much of the species' range (Wallace et al. 2011; Fuller et al. 2017). This increase in frequency is consistent with *D* < 0 when we sample only AR-assigned strains. In addition, when we compared AR with all other arrangements, we observed elevated F_ST_ within the inverted region (Fig. 5b). Elevated F_ST_ between inversion arrangements is expected for adaptively diverging arrangements (Guerrero et al. 2012).

Third chromosomes that we assigned to the TL arrangement were notable outliers, with Tajima's *D* > 0 (Fig. 3b). *D* was particularly elevated within and surrounding the location of the inversion that gave rise to the TL arrangement, consistent with previous results (Fuller et al. 2017). TL is one of the oldest inversion arrangements (Wallace et al. 2011), and TL chromosomes are rare across most of the species' range (Dobzhansky 1944; Anderson et al. 1991). If TL has decreased in frequency, it could harbor an excess of intermediate frequency alleles (Maruyama and Fuerst 1985), which would cause *D* > 0.

Our analyses of genome-wide SNPs in *D. pseudoobscura* both confirmed previous findings and uncovered new patterns that were not observed in prior work. For example, we confirmed inferences about the effects of demographic history and third chromosome inversion arrangements on genetic variation that were made in previous studies using fewer loci, fewer individuals, and no chromosome-scale reference. However, we also showed how polymorphic chromosomal inversions can be detected from signatures of population structure. Specifically, our work demonstrated how combining tests for population structure with analyses of genetic variation and differentiation can identify inversion. We additionally discovered additional outlier patterns of genetic variation on *D. pseudoobscura* chromosome 5 that should be examined in future work. The availability of our population genomics resource will facilitate such work.

## Supplementary Material

jkag210_Supplementary_Data

## Data Availability

All sequence data have been deposited in the NCBI SRA under BioProject accession PRJNA1413398. Additional data and analysis commands are available from dx. doi.org/10.18738/T8/QLQBOD. Supplemental material available at G3 online.
