# Supplementary material for: A population genomic resource for Drosophila pseudoobscura
Source: G3 (Bethesda). 2026 Aug 1;16(10):jkag210. doi: 10.1093/g3journal/jkag210 (PMC13643758; doi:10.1093/g3journal/jkag210)
Supplement: Supplementary file 1 [file jkag210_supplementary_data.zip › Supplemental_Figures_G3-2026-407110.pdf]

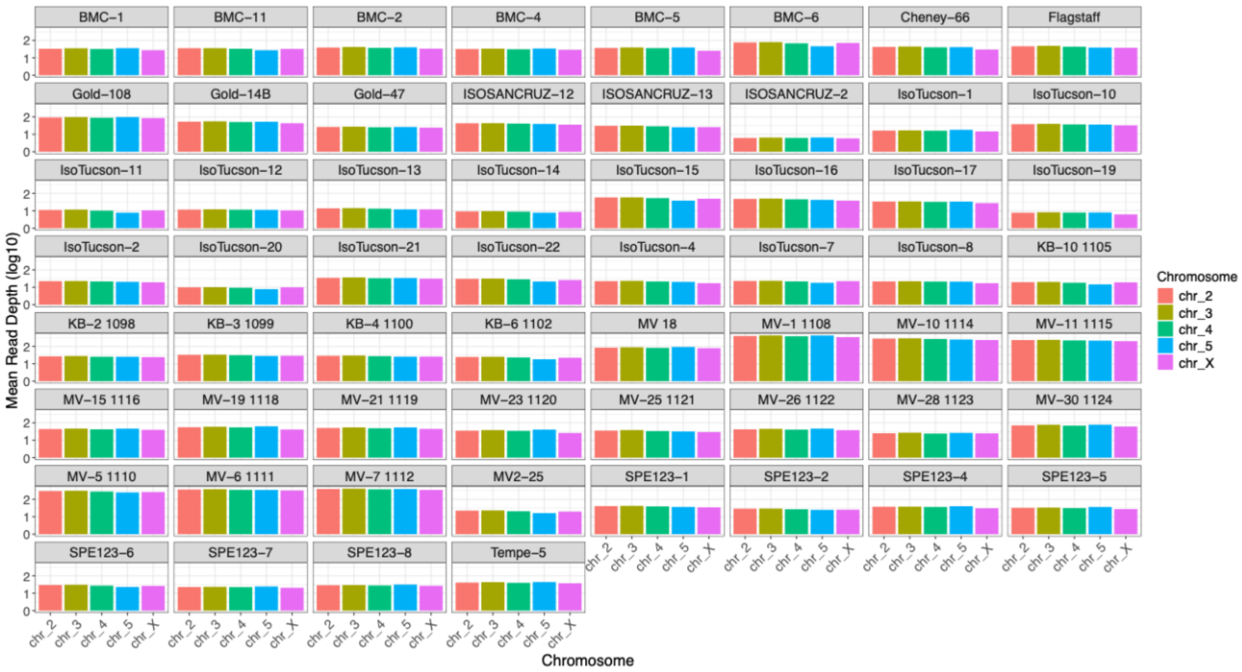

Supplementary Figure S1. Log10-transformed mean read depth across *Drosophila pseudoobscura* chromosomes for each strain.

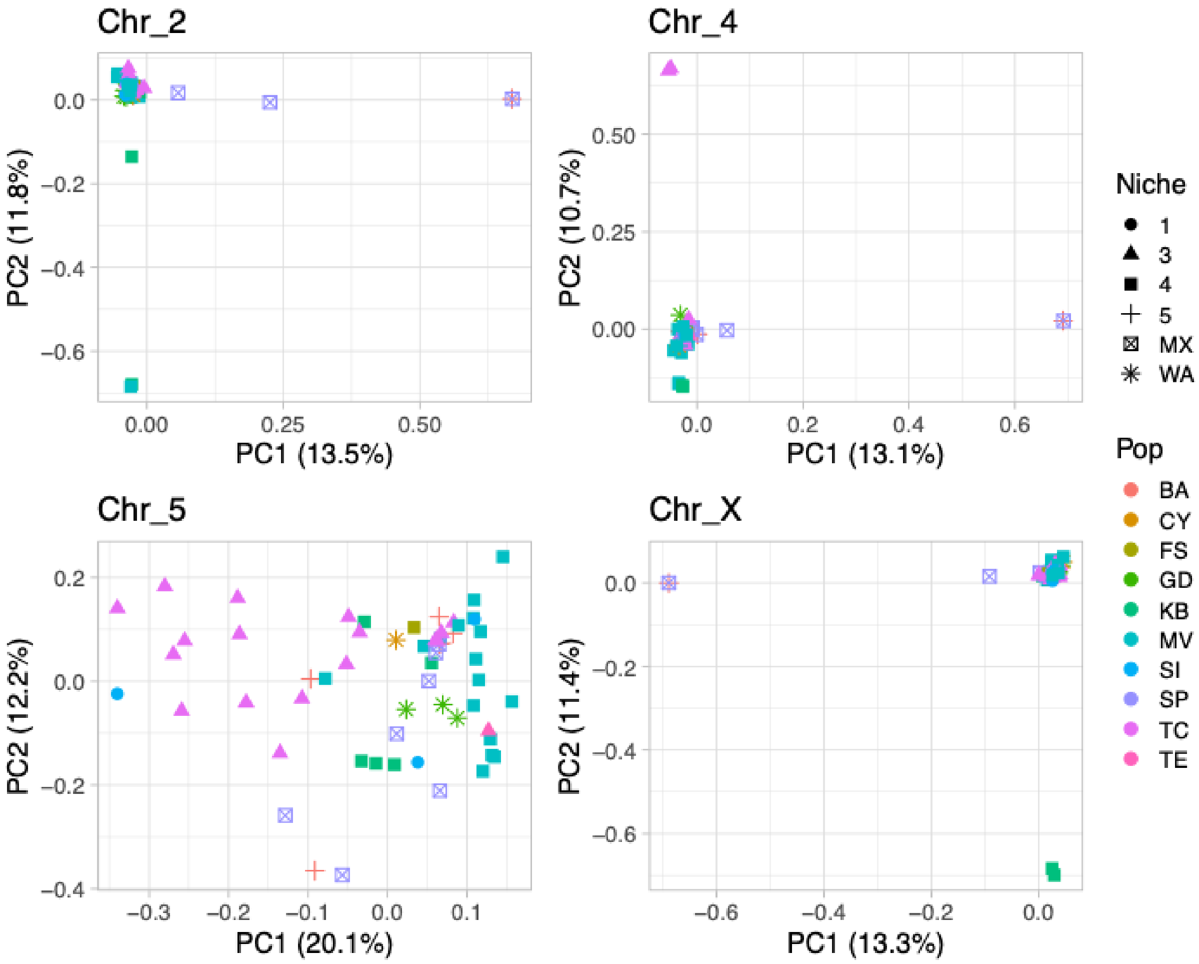

Supplementary Figure S2. Principal component analysis showing the first two principal components (PC1 and PC2) for *Drosophila pseudoobscura* chromosomes 2, 4, 5 and X. Points are colored by population (Pop) and shaped by niche.

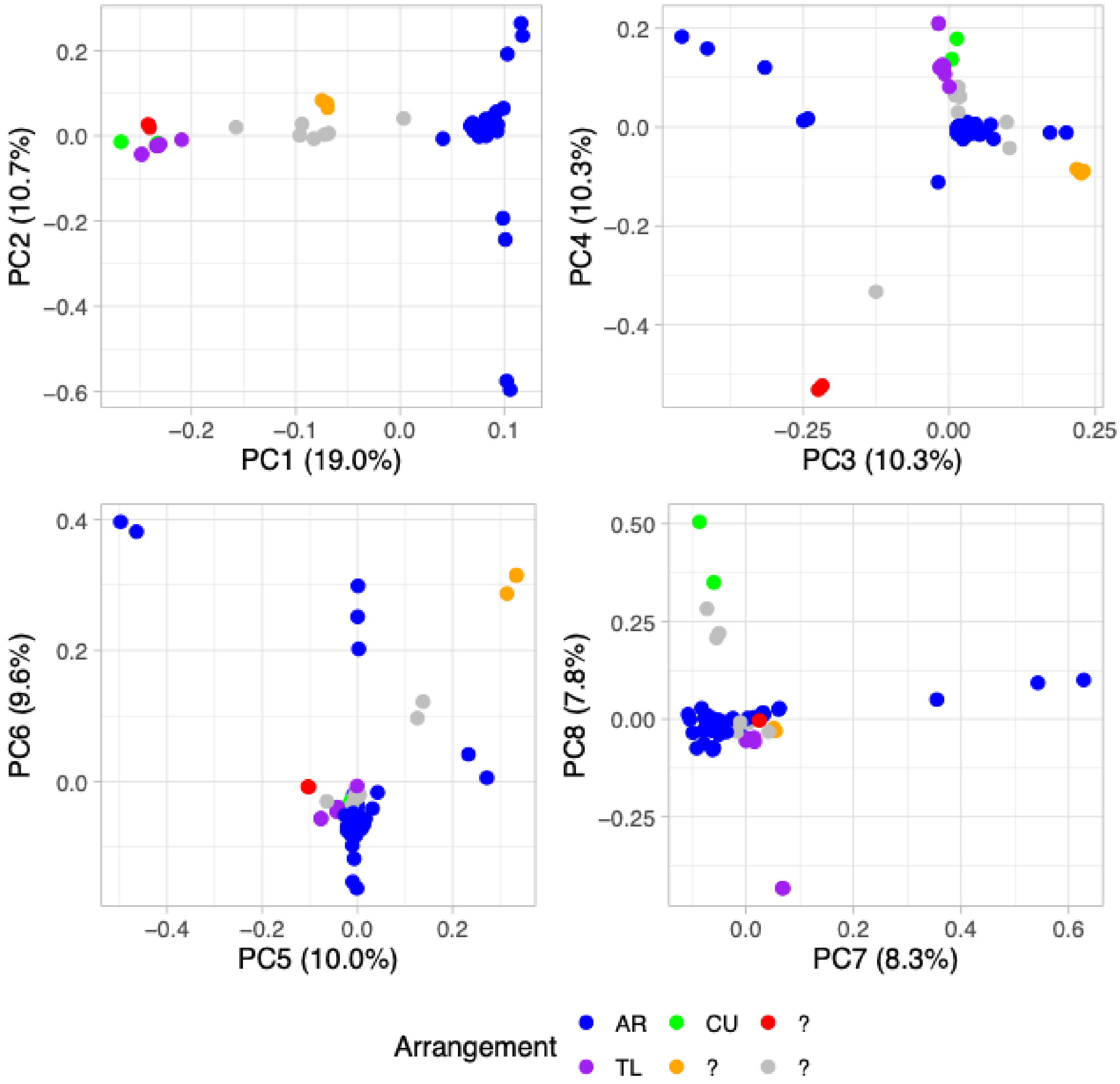

Supplementary Figure S3. Principal component analysis for *Drosophila pseudoobscura* chromosome 3. Scatterplots show pairs of principal components (PCs). Points are colored by chromosome 3 arrangement assignments (described in the main text).

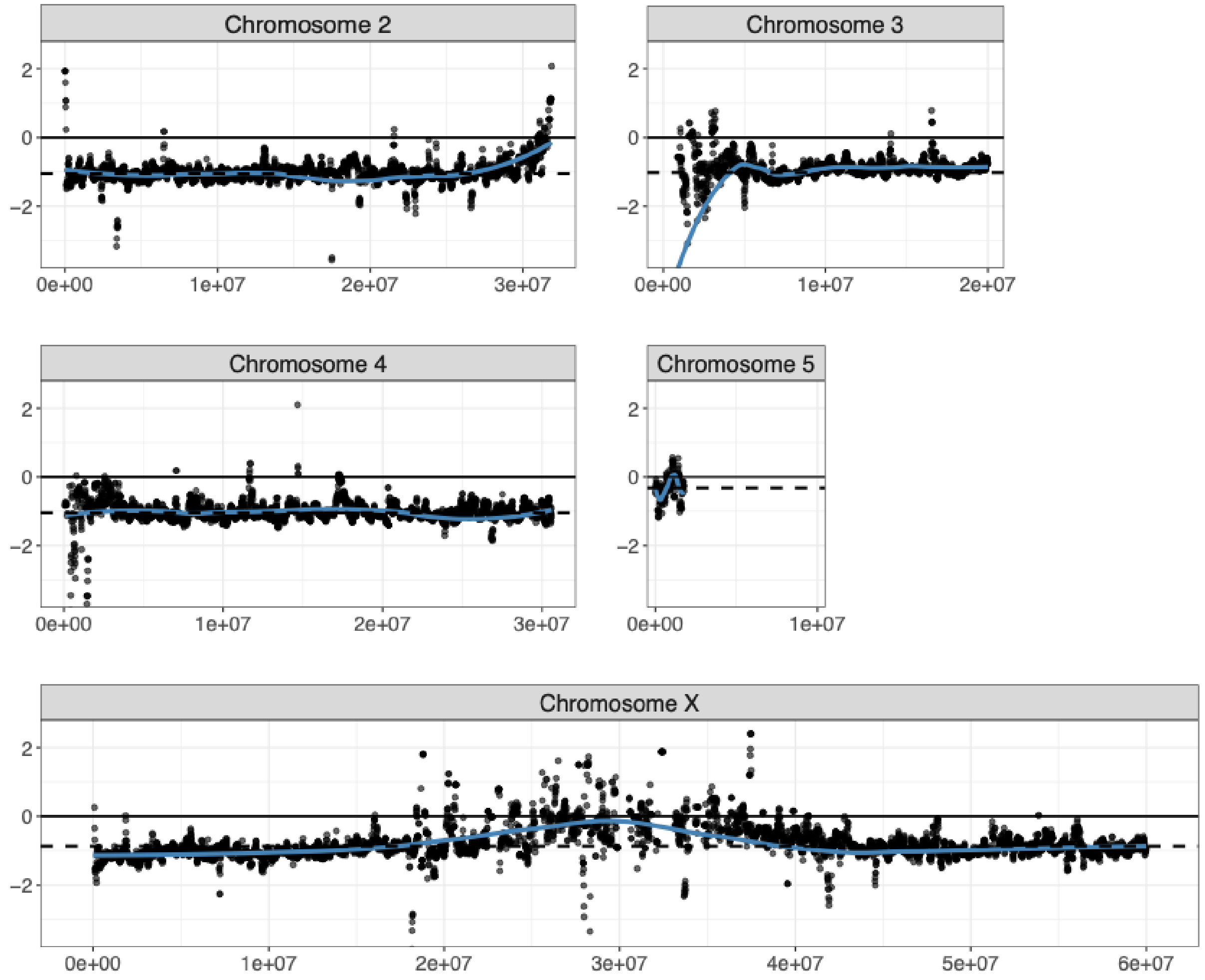

Supplementary Figure S4. Tajima's *D* across chromosomal windows using intronic sites in the *Drosophila pseudoobscura* genome. Black dots show Tajima's *D* values for 100 kp sliding windows (with a 10 kp step) across each of the five chromosomes. The solid blue lines show the generalized additive models with integrated smoothness estimation (GAMs) for each chromosome. The dashed black lines show the mean Tajima's *D* values for each chromosome.

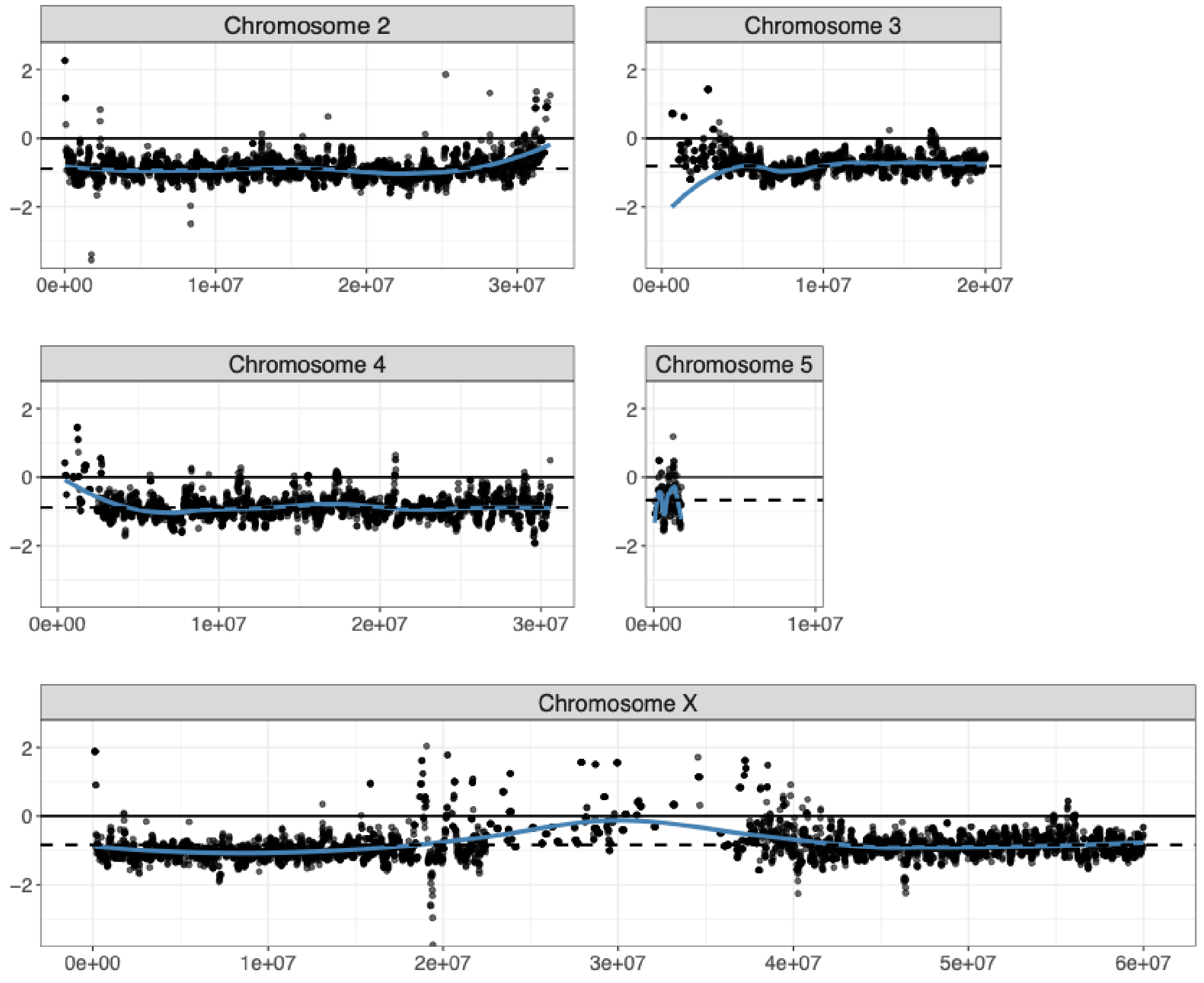

Supplementary Figure S5. Tajima's *D* across chromosomal windows using synonymous sites in the *Drosophila pseudoobscura* genome. Black dots show Tajima's *D* values for 100 kp sliding windows (with a 10 kp step) across each of the five chromosomes. The solid blue lines show the generalized additive models with integrated smoothness estimation (GAMs) for each chromosome. The dashed black lines show the mean Tajima's *D* values for each chromosome.

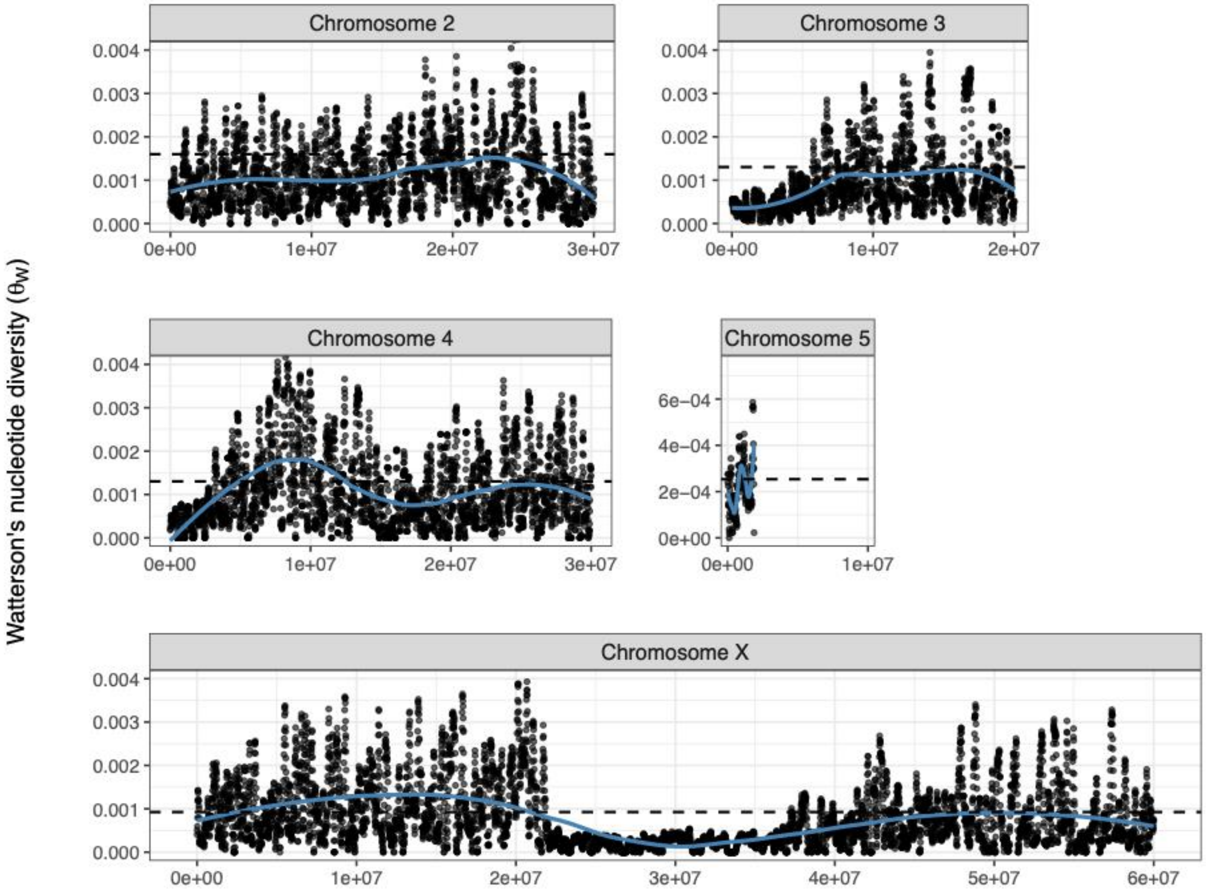

Supplementary Figure S6. Segregating genetic variation (Waterson's Θ_W) across windows on each *Drosophila pseudoobscura* chromosome. Black dots show Θ_W for 100 kb sliding windows (with a 10 kb step) using intronic sites across each of the five chromosomes arms. The solid blue lines show the generalized additive models with integrated smoothness estimation (GAMs) for each chromosome. The dashed black lines show the mean Θ_W for each chromosome.

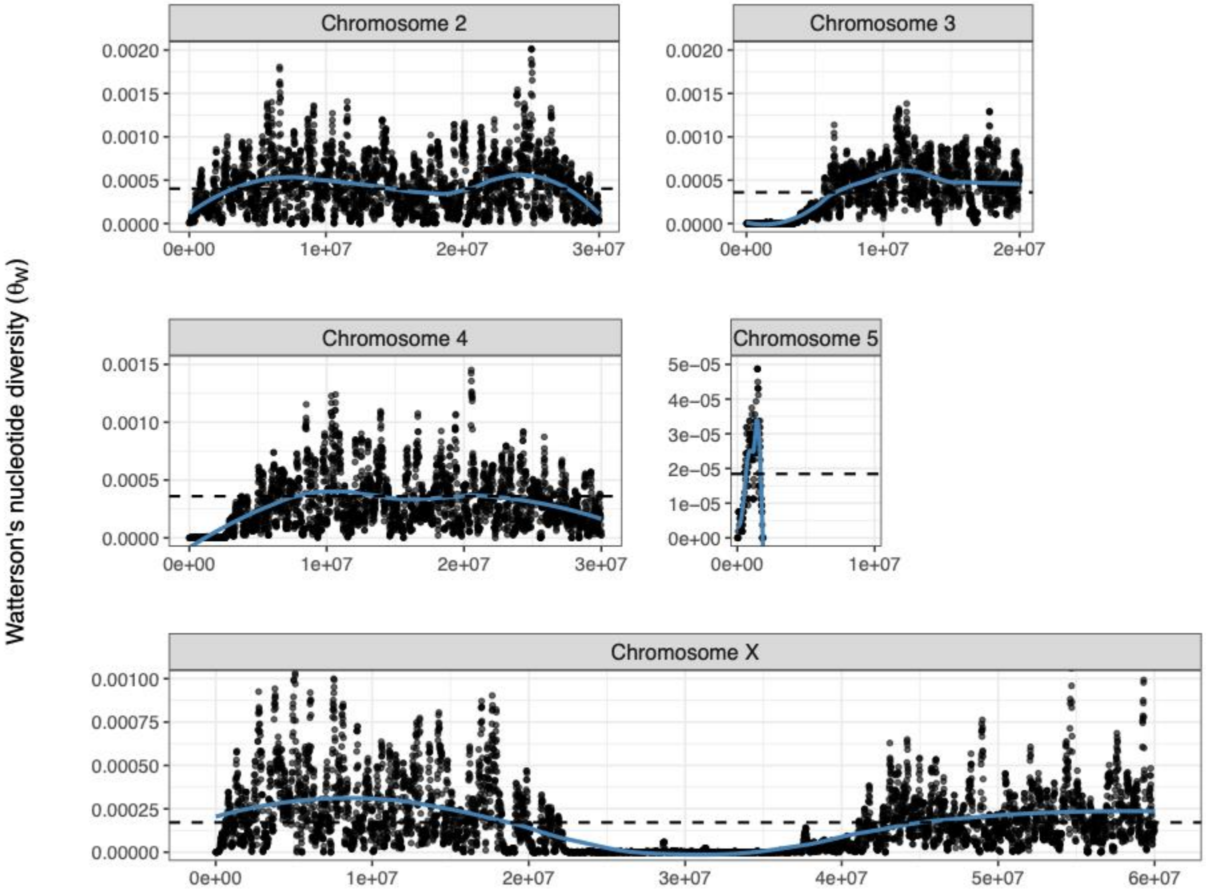

Supplementary Figure S7. Segregating genetic variation (Waterson's Θ_{W}) across windows on each *Drosophila pseudoobscura* chromosome. Black dots show Θ_{W} for 100 kb sliding windows (with a 10 kb step) using synonymous sites across each of the five chromosomes arms. The solid blue lines show the generalized additive models with integrated smoothness estimation (GAMs) for each chromosome. The dashed black lines show the mean Θ_{W} for each chromosome.

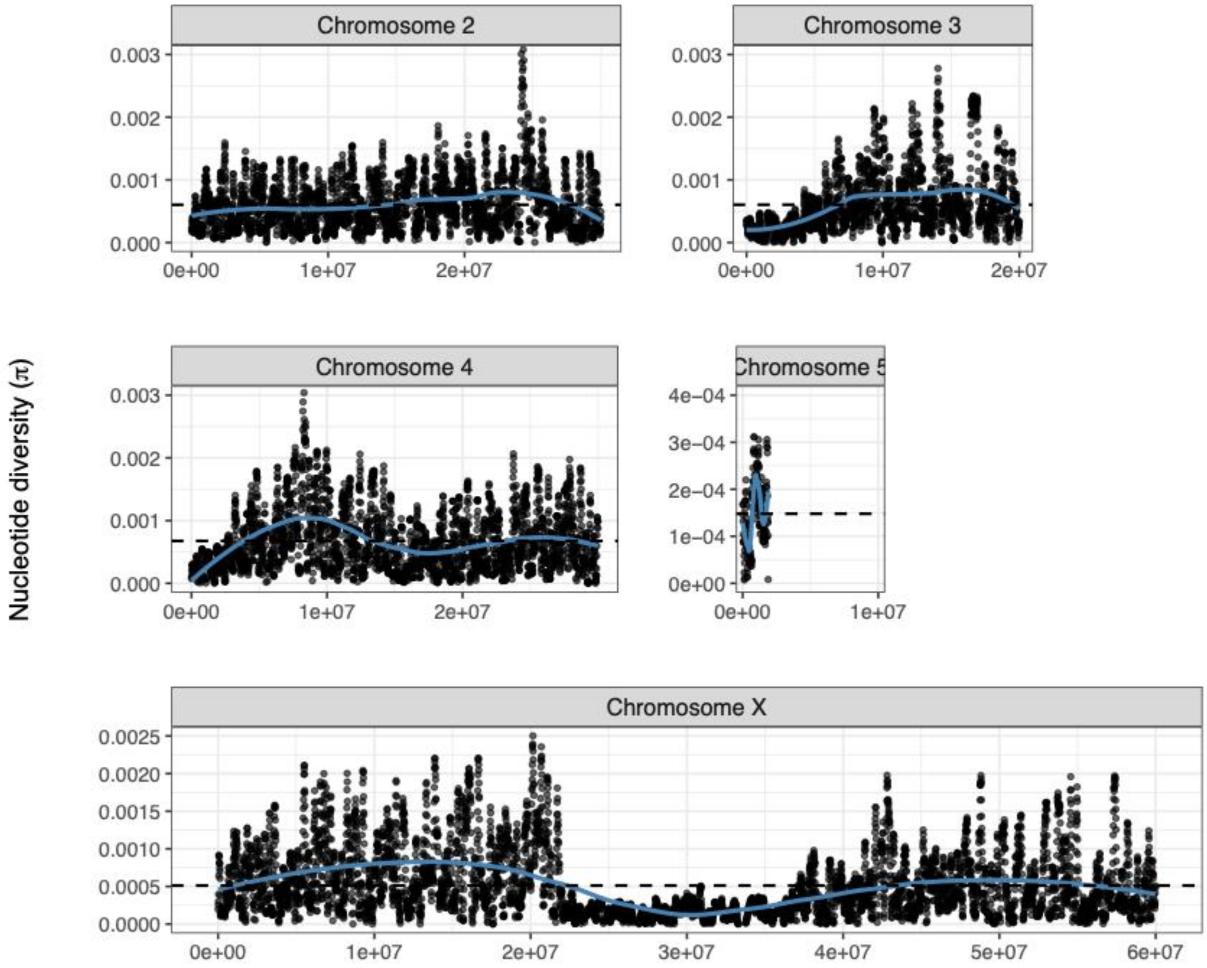

Supplementary Figure S8. Average pair-wise differences (π) across windows on each *Drosophila pseudoobscura* chromosome. Black dots show π for 100 kb sliding windows (with a 10 kb step) using intergenic sites across each of the five chromosomes arms. The solid blue lines show the generalized additive models with integrated smoothness estimation (GAMs) for each chromosome. The dashed black lines show the mean π for each chromosome.

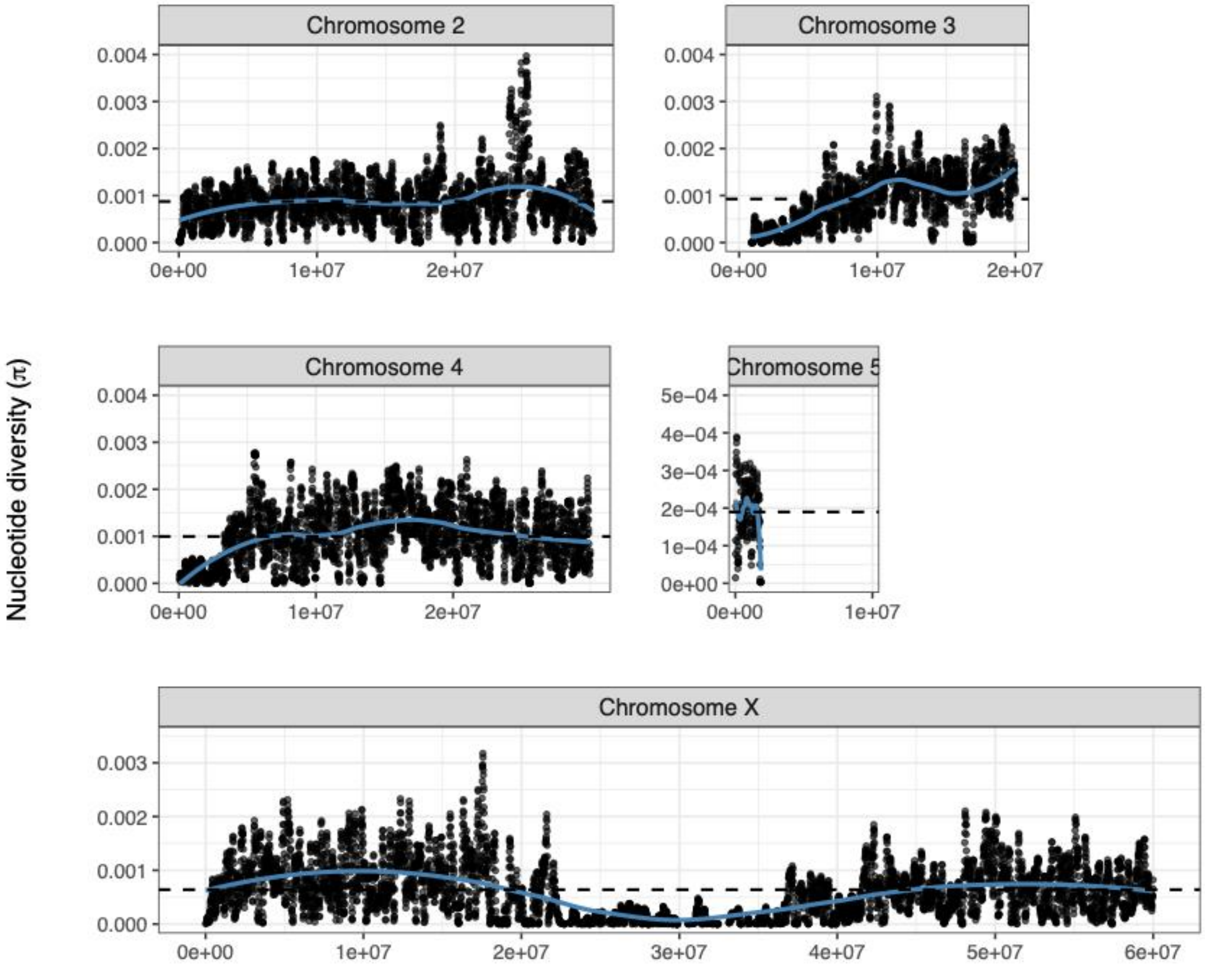

Supplementary Figure S9. Average pair-wise differences (π) across windows on each *Drosophila pseudoobscura* chromosome. Black dots show π for 100 kb sliding windows (with a 10 kb step) using intronic sites across each of the five chromosomes arms. The solid blue lines show the generalized additive models with integrated smoothness estimation (GAMs) for each chromosome. The dashed black lines show the mean π for each chromosome.

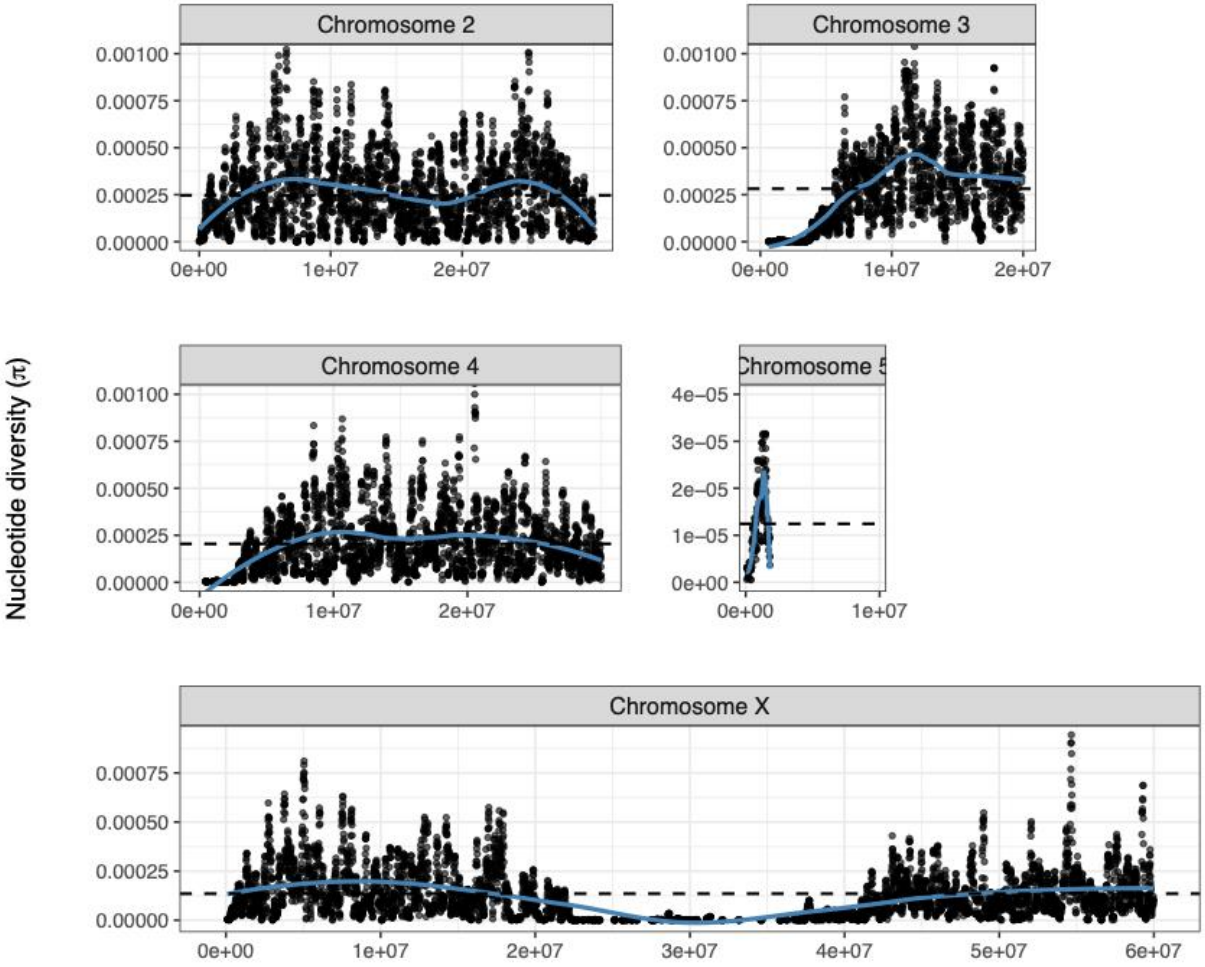

Supplementary Figure S10. Average pair-wise differences (π) across windows on each *Drosophila pseudoobscura* chromosome. Black dots show π for 100 kb sliding windows (with a 10 kb step) using synonymous sites across each of the five chromosomes arms. The solid blue lines show the generalized additive models with integrated smoothness estimation (GAMs) for each chromosome. The dashed black lines show the mean π for each chromosome.

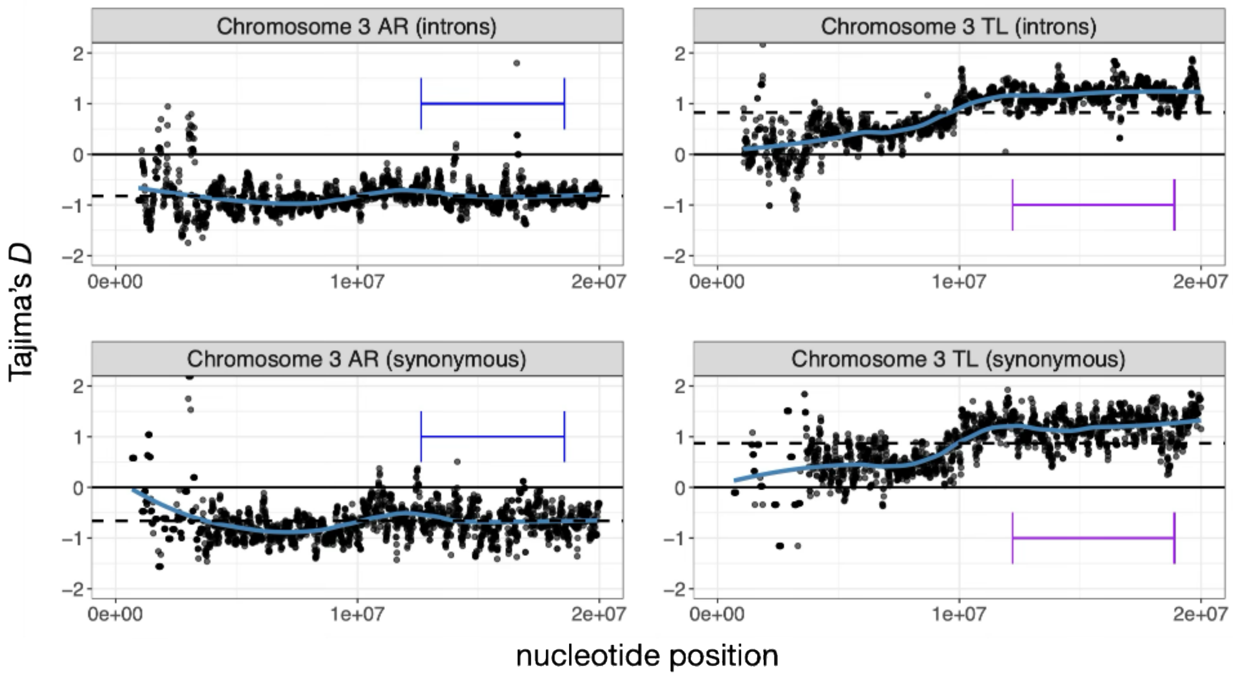

Supplementary Figure S11. Tajima's *D* across the *Drosophila pseudoobscura* third chromosome. Black dots show Tajima's *D* values for 100 kb sliding windows (with a 10 kb step) using intronic sites (top) or synonymous sites (bottom). Tajima's *D* was calculated using third chromosome genetic variation from either AR-assigned (left) or TL-assigned (right) strains. Blue and purple horizontal lines indicate the positions of the AR and TL inversions, respectively. The solid blue fit lines show the generalized additive models with integrated smoothness estimation (GAMs) for each chromosome. The dashed black lines show the mean Tajima's *D*.
