# Supplementary material for: A population genomic resource for Drosophila pseudoobscura
Source: G3 (Bethesda). 2026 Aug 1;16(10):jkag210. doi: 10.1093/g3journal/jkag210 (PMC13643758; doi:10.1093/g3journal/jkag210)
Supplement: Supplementary file 1 [file jkag210_supplementary_data.zip › Supplemental_Tables_S1-S3_G3-2026-407110.pdf]

Supplementary Tables for A population genomic resource for *Drosophila pseudoobscura*

Supplementary Table S1. List of 60 *D. pseudoobscura* strains used in this study. Strains were sampled from 10 geographically distinct populations across the species' range in North America. The four samples that did not pass filtering (see the Methods) are highlighted in red and labeled as "NO" in the "retained?" column.

sample number	strain	pop code	location	known arrangement	retained?
1	MV-1 1108	MV	Mesa Verde, CO	-	YES
2	MV-5 1110	MV	Mesa Verde, CO	-	YES
3	MV-6 1111	MV	Mesa Verde, CO	-	YES
4	MV-7 1112	MV	Mesa Verde, CO	-	YES
5	MV-10 1114	MV	Mesa Verde, CO	-	YES
6	MV-11 1115	MV	Mesa Verde, CO	-	YES
7	MV-15 1116	MV	Mesa Verde, CO	-	YES
8	MV 18	MV	Mesa Verde, CO	-	YES
9	MV-19 1118	MV	Mesa Verde, CO	-	YES
10	MV-21 1119	MV	Mesa Verde, CO		YES
11	MV-23 1120	MV	Mesa Verde, CO	-	YES
12	MV-25 1121	MV	Mesa Verde, CO	-	YES
13	MV-26 1122	MV	Mesa Verde, CO	-	YES
14	MV-28 1123	MV	Mesa Verde, CO	-	YES
15	MV-30 1124	MV	Mesa Verde, CO	-	YES
16	BMC-1	BA	Bosque de Apache NWF, NM	-	YES
17	BMC-2	BA	Bosque de Apache NWF, NM	-	YES
18	BMC-4	BA	Bosque de Apache NWF, NM	-	YES
19	BMC-5	BA	Bosque de Apache NWF, NM	-	YES
20	BMC-6	BA	Bosque de Apache NWF, NM	-	YES
21	BMC-10	BA	Bosque de Apache NWF, NM	-	NO
22	BMC-11	BA	Bosque de Apache NWF, NM	-	YES
23	BMC-12	BA	Bosque de Apache NWF, NM	-	NO
24	KB-2 1098	KB	Kaibab, AZ	-	YES
25	KB-3 1099	KB	Kaibab, AZ	-	YES
26	KB-4 1100	KB	Kaibab, AZ	-	YES
27	KB-5 1101	KB	Kaibab, AZ	-	NO
28	KB-6 1102	KB	Kaibab, AZ	-	YES
29	KB-10 1105	KB	Kaibab, AZ	-	YES
30	SPE123-1	SP	San Pablo Etla, MX	-	YES
31	SPE123-2	SP	San Pablo Etla, MX	TL	YES

sample number	strain	pop code	location	known arrangement	retained
32	SPE123-4	SP	San Pablo Etla, MX	CU	YES
33	SPE123-5	SP	San Pablo Etla, MX	TL	YES
34	SPE123-6	SP	San Pablo Etla, MX	TL	YES
35	SPE123-7	SP	San Pablo Etla, MX	-	YES
36	SPE123-8	SP	San Pablo Etla, MX	-	YES
37	MV2-25	MV	Mesa Verde, CO	-	YES
38	Flagstaff	FS	Flagstaff, AZ	-	YES
39	Gold-108	GD	Goldendale, WA	-	YES
40	Gold-14B	GD	Goldendale, WA	-	YES
41	Gold-47	GD	Goldendale, WA	-	YES
42	Tempe-5	TE	Tempe, AZ	-	YES
43	Cheney-66	CY	Cheney, WA	-	YES
44	IsoTucson-6	TC	Tucson, AZ	-	NO
45	IsoTucson-7	TC	Tucson, AZ	-	YES
46	IsoTucson-10	TC	Tucson, AZ	-	YES
47	IsoTucson-13	TC	Tucson, AZ	-	YES
48	IsoTucson-14	TC	Tucson, AZ	-	YES
49	IsoTucson-20	TC	Tucson, AZ	-	YES
50	IsoTucson-1	TC	Tucson, AZ	-	YES
51	IsoTucson-2	TC	Tucson, AZ	-	YES
52	IsoTucson-4	TC	Tucson, AZ	-	YES
53	IsoTucson-8	TC	Tucson, AZ	-	YES
54	IsoTucson-11	TC	Tucson, AZ	-	YES
55	IsoTucson-12	TC	Tucson, AZ	-	YES
56	IsoTucson-15	TC	Tucson, AZ	-	YES
57	IsoTucson-16	TC	Tucson, AZ	-	YES
58	IsoTucson-17	TC	Tucson, AZ	-	YES
59	IsoTucson-19	TC	Tucson, AZ	-	YES
60	IsoTucson-21	TC	Tucson, AZ	-	YES
61	IsoTucson-22	TC	Tucson, AZ	-	YES
62	ISOSANCRUZ-2	SI	Santa Cruz Island, CA	-	YES
63	ISOSANCRUZ-12	SI	Santa Cruz Island, CA	TL	YES
64	ISOSANCRUZ-13	SI	Santa Cruz Island, CA	-	YES

Supplementary Table S2. Number of SNPs per chromosome after initial filtering and number of SNPs retained after applying filtering criteria for population structure analysis. SNPs were retained if they had a genotype call rate of at least 95% (--max-missing 0.95) and a minor allele frequency of at least 5% (--maf 0.05) across the remaining strains.

Chromosome	Initial SNPs	Retained SNPs	Percent Retained
Chromosome 2	504,501	201,653	39.9%
Chromosome 3	351,536	157,367	44.8%
Chromosome 4	519,967	195,063	37.5%
Chromosome 5	5,396	1,840	34.1%
Chromosome X	723,865	275,885	38.1%
sum	2,105,265	831,808	39.5%

Supplementary Table S3. Population structure inferred by fastSTRUCTURE for each chromosome. The table shows the range of *K* values tested, along with the model complexity that maximized the marginal likelihood and the number of model components used to explain structure in the data under both the simple and logistic priors.

Chromosome	FastSTRUCTURE prior	*K* tested	Model complexity that maximizes marginal likelihood	Model components used to explain structure in data
2	Simple prior	*K*=1-10	2	2
3	Simple prior	*K*=1-10	5	5
4	Simple prior	*K*=1-10	1	2
	Logistic prior	*K*=1-2	2	1
5	Simple prior	*K*=1-10	9	7
	Logistic prior	*K*=1-4	9	5
X	Simple prior	*K*=1-10	1	3
	Logistic prior	*K*=1-3	3	1

Supplementary Table S4. List of genes found in overlapping troughs of Tajima's *D* between intergenic and intronic sites on chromosomes 3 and 4.

Due to its size, Supplementary Table S4 is provided as a separate comma-separated values (CSV) file (Table_S4.csv) rather than being included in the supplemental document.
